# Cytotoxic Compounds of Two Demosponges (*Aplysina aerophoba* and *Spongia* sp.) from the Aegean Sea

**DOI:** 10.3390/biom11050723

**Published:** 2021-05-12

**Authors:** Maria Orfanoudaki, Anja Hartmann, Mostafa Alilou, Naida Mehic, Marcel Kwiatkowski, Karin Jöhrer, Hieu Nguyen Ngoc, Andreas Hensel, Richard Greil, Markus Ganzera

**Affiliations:** 1 Institute of Pharmacy/Pharmacognosy, University of Innsbruck, Innrain 80-82, 6020 Innsbruck, Austria; orfmaria@gmail.com (M.O.); mostafa.alilou@uibk.ac.at (M.A.); naida.mehic@icloud.com (N.M.); markus.ganzera@uibk.ac.at (M.G.); 2 Functional Proteo-Metabolomics, Department of Biochemistry, University of Innsbruck, Innrain 80-82, 6020 Innsbruck, Austria; Marcel.Kwiatkowski@uibk.ac.at; 3 Tyrolean Cancer Research Institute, 6020 Innsbruck, Austria; karin.joehrer@tkfi.at (K.J.); r.greil@salk.at (R.G.); 4 Faculty of Pharmacy, Phenikaa University, Hanoi 12116, Vietnam; hieu.nguyenngoc@phenikaa-uni.edu.vn; 5 A&A Green Phoenix Group JSC, Phenikaa Research and Technology Institute (PRATI), No. 167 Hoang Ngan, Trung Hoa, Cau Giay, Hanoi 11313, Vietnam; 6 Institute of Pharmaceutical Biology and Phytochemistry, University of Münster, Corrensstraße 48, 48149 Münster, Germany; ahensel@uni-muenster.de; 7 Cancer Cluster Salzburg, IIIrd Medical Department, Paracelsus Medical University Salzburg, Müllner Hauptstraße 48, 5020 Salzburg, Austria

**Keywords:** *Spongia* sp., *Aplysina aerophoba*, brominated compounds, prenylated hydroquinones, cytotoxicity, antimicrobial agents

## Abstract

The class of demosponges is the biggest and most diverse of all described sponge species and it is reported to produce a plethora of chemically different metabolites with interesting biological activities. The focus of the present study was to investigate the chemical composition of two Mediterranean demosponges, targeting their brominated compounds and prenylated hydroquinones, compounds with interesting cytotoxic and anti-microbial properties. In order to gain a deeper insight into the chemical diversity of their metabolites and their activities, 20 pure secondary metabolites including new natural products were isolated from two different species (*Aplysina aerophoba* and *Spongia* sp.) using various chromatographic techniques. Their structures were confirmed by NMR and HRMS, revealing molecules with various chemical scaffolds, mainly prenylated hydroquinones from *Spongia* sp. and halogenated compounds from *Aplysina aerophoba,* including 5 novel natural products. The isolated compounds were investigated for their cytotoxic properties using 9 different cell lines, and especially one compound, 2,6-dibromo-4-hydroxy-4-methoxycarbonylmethylcyclohexa-2,5-dien-1-one showed good activities in all tested models.

## 1. Introduction

Sponges are a prolific source of bioactive natural compounds with unique structural features unprecedented in the terrestrial environment [1]. Demospongiae is their biggest and most diverse class [2], and they are known to produce a variety of chemically different metabolites, including terpenes, alkaloids, macrolides, peptides, betaines, ceramides, lipids, and halogenated compounds with potential interest regarding industrial and medical applications including antiviral, antitumor, antimicrobial, or generally cytotoxic properties. Therefore, they are of considerable biotechnological interest [3,4]. These compounds often possess multiple ecological functions, primarily the protection against predators, competitors for space, biofoulers, or opportunistic pathogenic microorganisms [5].

The sponge *Aplysina aerophoba* Schmidt, 1862 (Demospongiae, Verongida, Aplysinidae) is a common Mediterranean, photophilic species growing on stable substrates like rocks or rocky walls [6]. Its bright-yellow color is due to the highly labile pigment uranidine which undergoes polymerization when exposed to air [7]. It is known to harbor large amounts of bacteria and contains high concentrations of brominated alkaloids [8,9], mainly isoxazoline alkaloids, which after wound-induced bioconversion, are enzymatically cleaved to the lower molecular weight antibacterial compounds, showing 3,5 dibromotyrosin structure. These compounds protect the sponge from penetration of pathogenic bacteria [10,11]. The isolated brominated metabolites from *A. aerophoba* have been tested mainly for their cytostatic and antimicrobial activities, often showing moderate to strong effects [12,13]. Potential interest regarding industrial and medical applications has led to attempts for sponge cultivation and increased production of sponge biomass [14].

The genus *Spongia*, Linnaeus 1759, comprising of approximately 90 species, belongs to the family Spongidae of the order Dictyoceratida [15]. They have commercial value since they are harvested as natural bath sponges in the Mediterranean and the West Indies [16]. Sponges of the genus *Spongia* are unarmored, soft to firm, and compressible. They have a skeletal network of primary fibers, and they are a rich source of collagen proteins [16,17]. They mainly produce terpenes (sesquiterpene quinones, diterpenes, furanoterpenes, sesterterpenes), sterols, macrolides, and alkaloids [15]. Additionally, long chain lipid compounds may be considered as part of the chemical fingerprint of *Spongia* genus [15]. Regarding bioactivity, some of these metabolites, especially those with terpene structure, are anti-viral and antioxidant compounds, they act as immunomodulators, and they are active against different cancer cell lines [15,18].

The aim of the present study was the investigation of the chemical composition of two Mediterranean demosponges, *Aplysina aerophoba* and *Spongia* sp., targeting their brominated compounds and prenylated hydroquinones, compounds with interesting cytotoxic properties.

**Figure 1:**
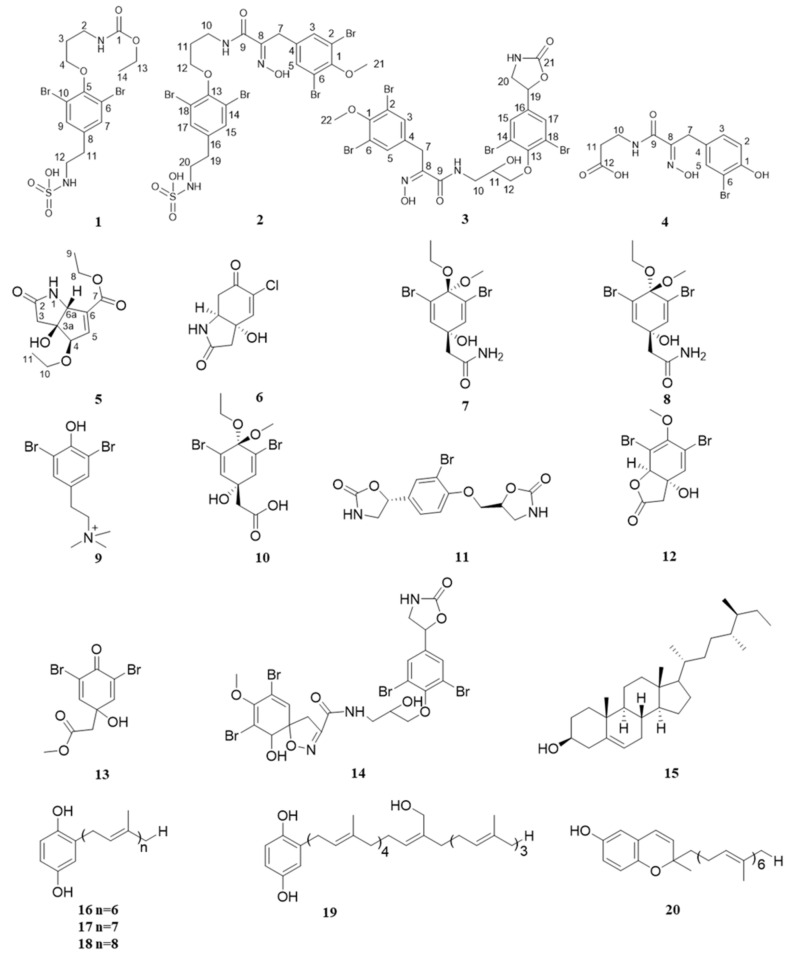
The chemical structures of compounds **1** to **20** as elucidated by NMR and MS; compounds **1**–**5** are novel natural products isolated from *A. aerophoba*, compounds **6**–**15** were isolated from *A. aerophoba* and they have been previously described in the literature, compounds **16**–**20** were isolated from *Spongia* sp. and they are known compounds.

Fifteen metabolites with diverse structures were isolated from the ethanolic extract of *A. aerophoba*, including two new chemical constituents for this species, five new natural products, and eight known ones. Additionally, five known compounds could be isolated from the ethanolic extract of *Spongia* sp. (Figure 1). The cytotoxic effects of the pure metabolites were evaluated in nine different cell lines, including a human urinary bladder carcinoma cell line (T24), a human stomach carcinoma cell line (AGS), a human neuroblastoma cell line (SH-SY5Y), three human colon adenocarcinoma cell lines (DLD-1, SW-480, LOVO), and three myeloma cell lines (NCI-H929, OPM-2, U266).

## 2. Materials and Methods

### 2.1. Biological Material

*Spongia* sp. was collected at Heraklion, Crete, Greece in April 2018, and *Aplysina aerophoba* at the same place in June 2018. Both were morphologically identified by Dimitris Poursanidis (Postdoc fellow, IACM-FORTH, Foundation for Research and Technology—Heraklion, Crete, Greece—CEO of terraSolutions marine environment research). The sponges were cut into small pieces and immediately stored in EtOH 96% until further processing. Voucher samples are deposited at the Institute of Pharmacy, Pharmacognosy, University of Innsbruck, Austria.

### 2.2. Instrumentation

Optical rotations were measured with a polarimeter P-2000 (JASCO, Tokyo, Japan) using a 10.0 cm tube and CHCl_3_ as the solvent. IR spectra were obtained on a Platinum ATR FTIR spectrometer (Bruker, MA, USA), and ECD experiments conducted on a J-1500 spectrophotometer (JASCO, Tokyo, Japan). NMR experiments were performed on two spectrometers from Bruker, Bruker Avance II 600 (600 MHz for ^1^H, 150 MHz for ^13^C) and Avance III HD (400 MHz for ^1^H, 100 MHz for ^13^C). The isolated compounds were dissolved in MeOD or chloroform using tetramethylsilane (TMS) as internal standard. High-resolution mass spectra were measured with aQ-Exactive HF-X Orbitrap mass spectrometer (Thermo, MA, USA) and a micrOTOF-Q II mass spectrometer (Bruker-Daltonics, Bremen, Germany) whereas low-resolution mass spectra were recorded on an Agilent InfinityLab LC/MSD System. It comprised of an Agilent 1260 HPLC, equipped with binary pump, autosampler, column oven, and photodiode array detector (Santa Clara, CA, USA). For the purification of compounds, a Reveleris^®^ X2 iES flash chromatography system (Büchi, Flawil, Switzerland) and a semi-preparative UltiMate 3000 HPLC from Dionex (Thermo, Waltham, MA, USA), comprising a P580 pump, an ASI 100 automated sample injector, an UVD 170 U detector, and a fraction collector, were used. Sephadex LH-20 material was purchased from Sigma-Aldrich (St. Louis, MI, USA). Analytical HPLC experiments were performed on a LC-20AD XR System (Shimadzu, Tokyo, Japan).

### 2.3. Chemicals and Reagents

All solvents required for extraction and isolation were purchased from VWR International (Vienna, Austria) and petroleum ether (PE), dichloromethane, acetone, and ethyl acetate (EtOAc) were distilled before use. Solvents for analytical experiments had pro analysis (p.a.) quality at least and were obtained from Merck (Darmstadt, Germany). Deuterated solvents were supplied by Euriso-Top (Saint-Aubin, France). Ultrapure water was produced by a Sartorius arium^®^ 611 UV (Göttingen, Germany) purification system. Silica gel 40–63 μm and pre-packed cartridges for flash chromatography were purchased from Merck (Darmstadt, Germany) and Büchi (Flawil, Switzerland), respectively.

### 2.4. Extraction and Isolation

*Aplysina aerophoba* (approximately 1 kg) was extracted five times in an ultrasonic bath (Bandelin Sonorex 35 KHz, Berlin, Germany) for 15 min each using EtOH 96%. Afterwards, the ethanolic extract (89 g) was partitioned successively between PE, EtOAc, BuOH, and H_2_O (3 times each × 500 mL). HPLC analysis of the fractions indicated that the PE (6.2 g), EtOAc (14.0 g), and BuOH (8.7 g) fractions contained brominated compounds, therefore, they were combined and used for further fractionation, while the water fraction (58.0 g) was dismissed.

**Figure 2:**
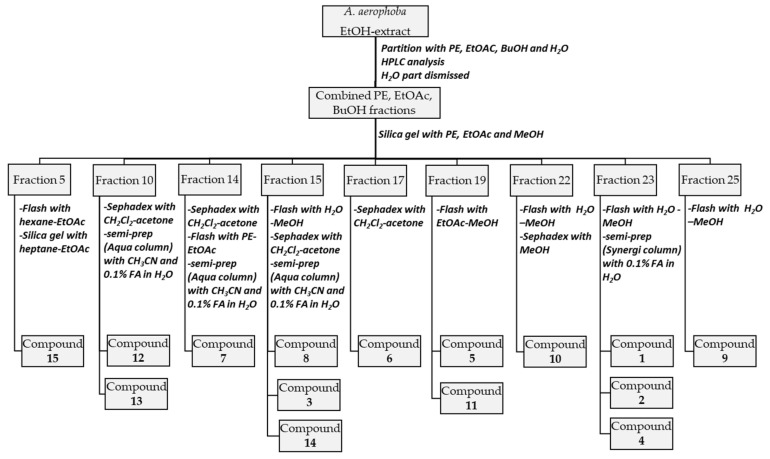
The isolation scheme for the *A. aerophoba* extract.

The combined PE, EtOAc, and BuOH fractions were fractionated on a silica gel column using PE, EtOAc, and methanol (from 10:0:0 to 0:10:0 to 0:0:10) as solvent, resulting in 27 subfractions (Figure 2). Fraction 5 (380 mg) was separated with flash chromatography using hexane-EtOAc (10:0–8:2) to give seven subfractions. Further purification of subfraction 6 (138 mg) on silica gel CC elution with heptane-EtOAc (10:0–8:2) yielded compound **15** (4 mg). Separation of fraction 10 (294 mg) by Sephadex LH-20 with CH_2_Cl_2_-acetone (85:15) resulted in 7 subfractions from which subfraction 7 was a pure compound (compound **12**, 130 mg). Subfraction 4 (61 mg) was subjected to semi-preparative HPLC using an Aqua C18 125 Å column (250 × 10 mm, 5 μm; Phenomenex, Torrance, CA, USA). The mobile phase comprised of CH_3_CN and 0.1% formic acid (FA) in water to give compound **13** (7 mg). Fraction 14 (3 g) was separated with Sephadex LH-20 using CH_2_Cl_2_-acetone (85:15) and flash chromatography with PE-EtOAc (10:0–0:10) and then purified by semi-preparative HPLC using the above-mentioned Aqua C18 column (CH_3_CN-0.1% FA in water) to yield compound **7** (58 mg). Fraction 15 (433 mg) was separated into 7 subfractions by flash chromatography with H_2_O-MeOH (10:0–0:10). Subfraction 7 (127 mg) was purified on Sephadex LH-20 material with CH_2_Cl_2_-acetone (85:15) and semi-preparative HPLC (Aqua C18 column, CH_3_CN-0.1% FA in water) to yield compound **8** (32 mg), whereas subfraction 6 (23 mg) was purified with semi-preparative HPLC (Aqua C18 column CH_3_CN-0.1% FA in water) to give compounds **3** (2 mg) and **14** (6 mg). Compound **6** (32 mg) was obtained from fraction 17 (224 mg) by Sephadex LH-20 with CH_2_Cl_2_-acetone (85:15), while compounds **5** (32 mg) and **11** (32 mg) were obtained from fraction 19 (389 mg) using flash chromatography with EtOAc-MeOH (10:0–0:10). Fraction 22 was purified by flash chromatography with H_2_O-MeOH (10:0–0:10) and on Sephadex LH-20 material with MeOH to give compound **10** (28 mg). From fraction 25 (1.6 g), compound **9** (260 mg) was obtained by flash chromatography (H_2_O-MeOH, 10:0–0:10). Finally, fraction 23 (1.4 g) was subjected to flash chromatography with H_2_O-MeOH (10:0–0:10) as eluent to obtain seven subfractions. Semi-preparative HPLC of subfraction 7 using a Synergi Polar-RP 80Å column (250 × 4.6 mm, 4 μm; Phenomenex, USA) and MeOH and 0.1% FA in water as mobile phase resulted in compounds **1** (5 mg) and **2** (7 mg), while by semi-preparative HPLC of the combined subfractions 5 and 6 using the same Synergi Polar-RP column, compound **4** (2 mg) could be obtained.

**Figure 3:**
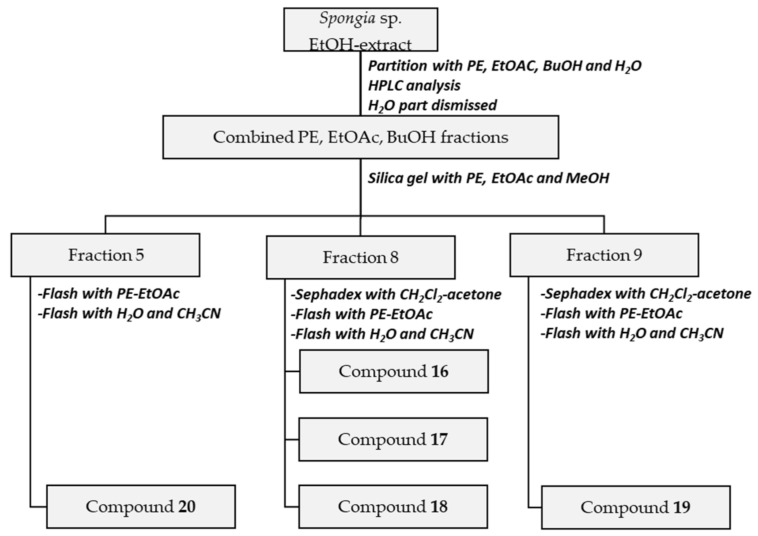
The isolation scheme for the *Spongia* sp. extract.

The ethanolic extract of *Spongia* sp. was fractionated using similar approaches as described above, resulting in the isolation of 5 compounds (Figure 3).

Initially, the material was extracted with EtOH 96% by sonication, the ethanolic extract (58 g) partitioned successively with PE, EtOAc, BuOH, and H_2_O (3 times each × 500 mL) and after HPLC analysis, the PE (5.7 g), EtOAc (2.5 g) and BuOH (4.7 g) soluble fractions combined, as results suggested the presence of prenylated hydroquinones.

The combined PE, EtOAc, and BuOH fractions were fractionated on a silica gel column using PE, EtOAc, and methanol (from 10:0:0 to 0:10:0 to 0:0:10), resulting in 23 subfractions. Fraction 9 (860 mg) was separated with Sephadex LH-20 with CH_2_Cl_2_-acetone (85:15) followed by flash chromatography using PE-EtOAc (10:0–0:10) to give 5 subfractions. Further purification of subfraction 4 (158 mg) via flash chromatography with H_2_O-CH_3_CN (10:0–0:10) yielded compound **19** (46 mg). Separation of fraction 8 (1.3 g) on Sephadex LH-20 material (CH_2_Cl_2_-acetone = 85:15 as eluent) resulted in 9 subfractions. Subfraction 7 was purified by normal phase flash chromatography with PE-EtOAc (10:0–0:10) and reversed phase flash chromatography with H_2_O-CH_3_CN (10:0–0:10) to give compounds **16** (71 mg) and **17** (520 mg), while subfraction 7 (410 mg) was subjected to reversed phase flash chromatography with H_2_O-CH_3_CN (10:0–0:10) resulting in compound **18** (14 mg). Finally, fraction 5 (300 mg) could be purified using flash chromatography with PE-EtOAc (10:0–0:10) and reversed phase flash chromatography with H_2_O-CH_3_CN (10:0–0:10) to yield compound **20** (8 mg).

#### 2.4.1. Compound **1**: Yellowish Amorphous Powder

R_f_ 0.70 (EtOAc:MeOH = 8:2); UV (MeOH) λ_max_ (log ε) 235 (3.7) nm; IR (KBr) ν_max_ 3286, 2933, 1687, 1540, 1456, 1252, 1171, 1033, and 775 cm^−1^; ^1^H and ^13^C NMR see Table 1; HR-ESI-MS *m/z* 500.9340 [M-H]^−^ (calcd. for C_14_H_19_Br_2_N_2_O_6_S, 500.93250).

#### 2.4.2. Compound **2**: Colourless Amorphous Powder

R_f_ 0.33 (EtOAc:MeOH = 8:2); UV (MeOH) λ_max_ (log ε) 236 (4.4) nm; IR (KBr) ν_max_ 3236, 3061, 2930, 1657, 1628, 1541, 1471, 1455, 1420, 1396, 1256, 1199, 1021.9, 1001, 866, and 737 cm^−1^; ^1^H and ^13^C NMR see Table 2; HR-ESI-MS *m/z* 775.7943 [M-H]^−^ (calcd. for C_21_H_22_Br_4_N_3_O_7_S, 775.7906).

#### 2.4.3. Compound **3**: Yellowish Amorphous Powder

R_f_ 0.82 (EtOAc:MeOH = 8:2); ${\left[\alpha \right]}_{D}^{21}$ −13.2 (c 0.9, CHCl_3_); UV (acetonitrile) λ_max_ (log ε) 236 (4.0) nm; IR (KBr) ν_max_ 3313, 2926, 2854, 1744, 1662, 1542, 1471, 1422, 1397, 1338, 1258, 1080, 1000, 939, and 739 cm^−1^; ^1^H and ^13^C NMR see Table 2; HR-ESI-MS *m/z* 753.8063 [M-H]^−^ (calcd. for C_22_H_20_Br_4_N_3_O_7_, 753.8059).

#### 2.4.4. Compound **4**: Yellowish Amorphous Powder

R_f_ 0.67 (EtOAc:MeOH = 8:2); UV (MeOH) λ_max_ (log ε) 236 (3.8) nm, 284 (3.5) nm; IR (KBr) ν_max_ 3280, 2942, 2834, 1713, 1659, 1532, 1508, 1494, 1366, 1283, 1207, 1016, 930, 801, and 671 cm^−1^; ^1^H and ^13^C NMR see Table 1; HR-ESI-MS *m/z* 366.9912 [M+Na]+ (calcd. for C_12_H_13_BrN_2_NaO_5,_ 366.9906).

#### 2.4.5. Compound **5**: Yellowish Amorphous Powder

R_f_ 0.78 (EtOAc:MeOH = 8:2); ${\left[\alpha \right]}_{D}^{21}$ −21.8 (c 0.1, CHCl_3_); UV (MeOH) λ_max_ (log ε) 235 (3.8) nm; IR (KBr) ν_max_ 3338, 2979, 2933, 2252, 1691, 1445, 1404, 1373, 1342, 1237, 1185, 1093, 1066, 764, and 706 cm^−1^; ^1^H and ^13^C NMR see Table 3; HR-ESI-MS *m/z* 256.1166 [M+H]^+^ calcd for C_12_H_18_NO_5_, 256.1185, 533.2119 [2M+H]^+^ calcd. for C_24_H_34_N_2_O_10_Na, 533.2111.

### 2.5. Computational Methods

The 3D structures of the molecules were drawn and subjected to conformational analysis in MacroModel v. 9 (Schrödinger LLC, New York, NY, USA) using OPLS-3 forcefield in chloroform. The conformers obtained in an energy window of 10 Kcal/mol were further submitted to geometrical optimization at B3LYP/6-31G(d,p) level for compounds **3** and **14**, and B3LYP/6-31++(d,p) for compound **5**. Further NMR chemical shift calculation was performed by using mPW1PW91/6-31G+(d,p)/CPCM/methanol for compounds **3** and **14** (data are not shown), and specific rotation calculation of compound **5** was conducted at B3LYP/6-311G++(d,p)/CPCM level in chloroform by considering the sodium D line frequency in the calculation. The obtained specific rotation values were Boltzmann-averaged and utilized for comparison with the experimentally obtained value in chloroform.

### 2.6. Cell Cultures

T24 cell line (ATCC HTB-4), derived from human urinary bladder carcinoma were obtained from Prof. Dr. Straube, University of Jena, GermanyT24 cells were cultured in Dulbecco’s modified Eagle medium (Merck Millipore, Darmstadt, Germany), supplemented with 10% (*v/v*) FCS (Merck) and 0.5% penicillin/streptomycin (Merck) at 5% CO_2_/37 °C. Passaging took place at 80 to 90% confluence of T24 cells.

The AGS cell line (300408, human stomach adenocarcinoma) were obtained from CLS Cell Lines Service GmbH and cells were cultured in RMPI-1640 (Merck), supplemented with 10% (*v/v*) FCS (Merck) and 1% penicillin/streptomycin (Merck) at 5% CO_2_/37 °C. Passaging took place at 80 to 90% confluence of AGS cells.

SH-SY5Y human neuroblastoma cell line was kindly provided by Dr. Obexer (Tyrolean Cancer Research Institute); human colon adenocarcinoma cell lines DLD-1, SW-480, and LOVO were purchased from DSMZ (Braunschweig, Germany). Human myeloma cell lines NCI-H929, OPM-2, and U266 were also purchased from DSMZ (Braunschweig, Germany). Cell lines were routinely fingerprinted and tested for mycoplasma negativity. Primary human foreskin fibroblasts were purchased from Promocell, Heidelberg, Germany.

PBMCs (peripheral blood mononuclear cells) from healthy donors were utilized after obtaining written consent at the University Hospital Salzburg (Ethics Committee approval 415-E/1287/6-2011). Cells were subjected to Ficoll separation (Ficoll Paque^TM^, VWR, Darmstadt, Germany). All cell lines and primary cells were grown in RPMI-1640 (Life Technologies, Paisley, UK), supplemented with 10% fetal calf serum (PAA, Linz, Austria), L-glutamine 100 µg/mL (Biochrom, Berlin, Germany), and penicillin-streptomycin 100 U/mL (Applichem, Darmstadt, Germany).

### 2.7. Determination of Cell Viability of T24 and AGS Cell Lines (MTT Assay)

To determine the influence of the isolated substances on cell viability towards T24 bladder and AGS stomach cells, MTT assay was performed (Mosmann, 1983). T24 cells were seeded into 96-well plates with 2.0 × 10^4^ cells per well (100 μL), incubated for 24 h at 37 °C with 5% CO_2_, and washed with 200 μL/well of PBS. Incubation of the cells with 100 μL of extract or pure substances at different concentrations (100 to 0.1 µM for pure compounds and 500–100 µg/mL for extracts in DMEM without additives) was performed for 24 h at 37 °C/5% CO_2_. Subsequently, the supernatant was removed and cells were washed twice with PBS (200 μL/well). In addition, 50 μL of MTT reagent were added to each well and after an incubation period of 24 h at 37 °C/5% CO_2_, the MTT reagent was removed and replaced by 50 µL DMSO per well to dissolve the formed insoluble formazan crystals. After 10 min, the amount of formazan was quantified spectrophotometrically in a plate reader at λ = 595 nm, with λ = 690 nm as a reference wavelength. Medium + 10% FCS served as a positive control, while the respective medium used for the sample preparation served as an untreated control. As a negative control, 10% DMSO was used. For AGS, a cell density of 5 × 10^4^ was used and substances were diluted in RPMI medium. At least three analyses in triplicates were performed for each cell line and each concentration of the compounds tested and a solvent control was always included. Data are shown as mean percentage of viable cells and standard error of the mean (SEM) (error bars).

### 2.8. Cytotoxicity Assays Using FACS Analysis

The induction of apoptosis was measured in cancer cell lines and in fibroblasts/peripheral blood mononuclear cells of healthy donors using established protocols [19]. Adherent cell lines (neuroblastoma cell line SH-SY5Y, colon carcinoma cell lines DLD-1, SW-480, and LOVO, as well as primary foreskin fibroblasts) were seeded at a concentration of 1 × 10^5^ cells/mL in 96-well plates the day before treatment in order to allow attachment. This medium was then replaced by media containing the respective compounds at different concentrations and cells were incubated for 24 h. Solvent controls were always included. Supernatants were collected and pooled with the trypsinized cells of the respective wells. These samples were then stained with AnnexinV-FITC (MabTag GmbH, Friesoythe, Germany) and propidium iodide (Merck, Darmstadt, Germany) and processed by flow cytometry using FACS Canto II and Diva software (Becton Dickinson, San Jose, CA, USA) Biosciences). Data were further analyzed using GraphPad Prism 5.0 software.

Myeloma cell lines NCI-H929, OPM-2, and U266 as well as peripheral blood mononuclear cells (PBMCs) of healthy donors (5 × 10^5^ cells/mL) were incubated with the same compounds for 24 h and subjected to FACS analysis as described above. Etoposide (Merck, Darmstadt, Germany) was used as positive control for adherent cancer cell lines and Bortezomib (Eubio, Vienna, Austria) was used as positive control for myeloma cell lines. The extent of non-apoptotic cells (AnnexinV/propidium iodide negativity) was calculated as percentage of control (untreated) and mean percentage of viable cells and standard deviation (error bars) are shown.

## 3. Results

### 3.1. Structure Elucidation

Five out of fifteen compounds isolated from *A*. *aerophoba* were novel natural products and their structure elucidation is described below.

#### 3.1.1. Compound **1**

**Figure 4:**
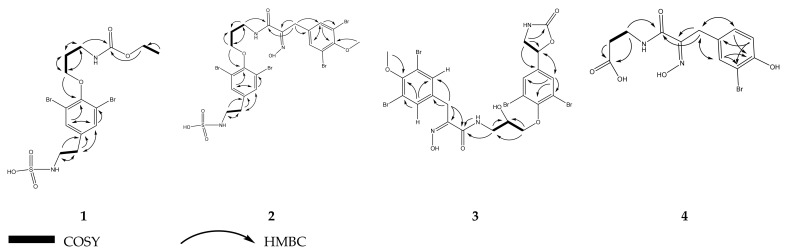
Key HMBC (^1^H → ^13^C) and ^1^H-^1^H COSY correlations of compounds **1**–**4**.

This substance was assigned to the molecular formula C_14_H_20_Br_2_N_2_O_6_S as established by a negative ion [M-H]^−^ at *m/z* 500.9340 (calcd. for C_14_H_19_Br_2_N_2_O_6_S 500.9325) in HR-ESI mass spectrum. Characteristic NMR chemical shifts (*δ*_H_ 7.49 for H-7, 9 and *δ*_C_ 152.9, 119.0, 134.5, 140.9 for C-5, 6, 7, 8, respectively) and MS patterns indicated the presence of a di-bromo substituted phenolic ring. The COSY spectrum revealed three coupling networks (Figure 4), including the protons of the methylenes H-2/H-3/H-4 and H-11/H-12 and the protons of the ethoxy group H-13/H-14. The substructures were connected through a carbamic acid bond by specific chemical shifts of position 1 (*δ*_C_ 159.3), 2 (*δ*_H_ 3.38, *δ*_C_ 39.2), and 13 (*δ*_H_ 4.07, *δ*_C_ 61.8) and key HMBC correlations of H-2, 13/C-1 (*δ*_C_ 159.2). Additionally, the HMBC correlations of H-4 (*δ*_H_ 4.02) to C-5 (*δ*_C_ 152.9) and H-11 and 12 (*δ*_H_ 2.81 and 3.19) to C-8 (*δ*_C_ 140.8) revealed the substitution of the phenolic ring (Figure 4). Specific chemical shifts of position 12 (*δ*_H_ 3.19, *δ*_C_ 46.2) as well as the suggested by HR-MS molecular formula revealed the existence of the sulfamic acid group. The sulfamic group is relatively rare in nature, however it has been found in sponges, already, for example, in araplysillin N-sulfamate isolated from *A. fulva* [20]. Compound **1**, named aeroplysinin-3, was finally identified as (3,5-dibromo-4-(3-((ethoxycarbonyl)amino)propoxy)phenethyl)sulfamic acid, a new natural product.

#### 3.1.2. Compound **3**

Compound **3** was assigned to the molecular formula C_22_H_21_Br_4_N_3_O_7_, determined by HR-ESI-MS (753.8063 calcd. for [M-H]^-^, found 753.8059). The NMR data revealed a new compound bearing the substructure of the right half of fistularin 1 [21], including a 2-oxazolidone ring joined directly to 2,6-dibromophenol, and a N-(2-hydroxypropyl)formamide moiety. Characteristic NMR chemical shifts indicated the presence of an additional 2,6-dibromo-4-methylene-phenol group, which was methylated at position 1, as indicated by the HMBC correlation of the methoxy group (*δ***_H_** 3.83) to C-1 (*δ***_C_** 153.0) (Figure 4). Furthermore, the protons of the methylene group H-7 (*δ***_H_** 3.89) showed an HMBC correlation to the carbon of an oxime group, C-8 (*δ***_C_** 152.6), and a further correlation with the carbon of the amide group of the first substructure, C-9 (*δ***_C_** 163.5). The IR spectrum also showed characteristic vibrational frequencies of N-O and C=N bonds of oxime group at 939 and 1662 cm^−1^. Compound **3** was finally identified as (*Z*)-N-(3-(2,6-dibromo-4-(2-oxooxazolidin-5-yl)phenoxy)-2-hydroxypropyl)-3-(3,5-dibromo-4-methoxyphenyl)-2-(hydroxyimino)propanamide, a new natural product with the trivial name aeroplysinin-5. In order to establish the absolute configuration of compounds **3** (and also its known derivative, **14**), one needs to decipher the relative stereochemistry prior to the absolute one. Compound **3** has chiral centers at C-11 and C-19. Due to the lack of NOEs between the protons of the respective carbon atoms, arising from being in two distant tails of the molecule, it was not possible to deduce any conclusion regarding their relative configuration. In an attempt to solve this issue, NMR chemical shift calculation along with computing of the DP4+ probability were applied on all the generated conformers of the possible stereoisomers of compound **3**. However, the obtained results failed to establish the relative configurations of the chiral centers, which is possibly arising from (i) the lack of interaction or effect between the two chiral centers, (ii) the high flexibility of the molecule which makes it difficult for proper conformational sampling. The process was similarly applied for the other known derivate (compound **14**), but no results could be concluded regarding its relative or absolute configuration, too.

#### 3.1.3. Compound **2**

Compound **2** was assigned to the molecular formula C_21_H_23_Br_4_N_3_O_7_S as established by a negative [M-H]^−^ signal at *m/z* 775.7943 (calcd. for C_21_H_22_Br_4_N_3_O_7_S 775.7906) in HR-ESI-MS. Characteristic NMR shifts indicated high similarity with compound **3**. However, three differences could be observed; the absence of oxygen at the second carbon of the propanol group, which was indicated by lower chemical shifts of the methylene of position 11 (*δ*_H_ 2.06, *δ*_C_ 30.6), COSY correlations of the methylenes H-10/H-11/H-12 (Figure 4), and the presence of an ethylsulfamic acid group instead of the oxazolidin-2-one ring attached to carbon of position 16. The latter was suggested by COSY correlations of the protons of methylenes H-19/H-20 and key HMBC correlations of H-20/C-16 (*δ*_C_ 140.0) and H-9/C-15, 17 (*δ*_C_ 134.5). Compound **2** named aeroplysinin-4, was finally identified as (*Z*)-(3,5-dibromo-4-(3-(3-(3,5-dibromo-4-methoxyphenyl)-2-(hydroxyimino)propanamido)propoxy)phenethyl)sulfamic acid, a new natural product.

#### 3.1.4. Compound **4**

**Figure 5:**
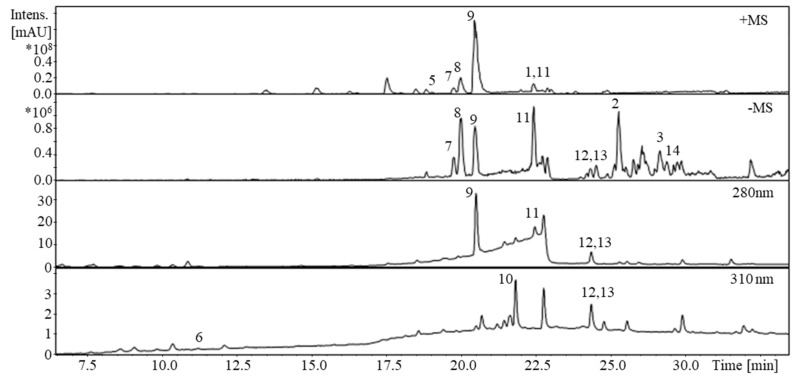
HPLC-UV-MS separation of the *A. aerophoba* extract. Peak assignment is according to Figure 1. Stationary phase: Synergi Polar-RP 80A (250 × 4, 6 mm; 4 μm) from Phenomenex (Torrance, CA, USA); mobile phase: 0.1% (*v/v*) FA in water (A) and CH_3_CN (B); Gradient: 0 min: 2% B, 10 min: 15% B, 18 min: 50% B, 30–35 min: 98% B, 35.1–45 min: 2% B; λ = 210, 254, 280, 310, and 350 nm; flow rate = 0.7 mL/min; T = 22 °C.

Compound **4** was assigned the molecular formula C_12_H_13_BrN_2_O_5_ as established by a positive ion [M+Na]^+^ at *m/z* 366.9912 (calcd. for C_12_H_13_BrN_2_NaO_5_ 366.9906). NMR shifts indicated the presence of a 2-bromo-4-methylenyl phenol moiety. The methylene group of the phenol ring was connected to a 2-(hydroxyimino) acetamide group as evidenced by specific chemical shifts of position 7 (*δ*_H_ 3.78, *δ*_C_ 28.8) and HMBC correlations of H-7/C-8, 9 (*δ*_C_ 153.1, 165.8). The presence of an oxime group was further supported by characteristic IR absorption bands at 930 and 1659 cm^−1^, which were assigned for N-O and C=N bonds. Furthermore, the COSY correlation of two methylenes, H-10 and H-11, as well as the HMBC correlations of H-11 (*δ*_H_ 2.48) to a carboxylic group at *δ*_C_ 176.9 and of H-10 (*δ*_H_ 3.48) to C-9, 12 (*δ*_C_ 165.8 and 176.9), revealed the chain attached to the NH group (Figure 4). Compound **4** was not present in the sponge’s extract (Figure 5), thus it is an artifact produced during the isolation procedure. Collectively, compound **4** named nor-psammaplin M was finally identified as (Z)-3-(3-(3-bromo-4-hydroxyphenyl)-2-(hydroxyimino)propanamido)propanoic acid, a new natural product.

#### 3.1.5. Compound **5**

Compound **5** was assigned the molecular formula C_12_H_17_NO_5_ as established by two typical signals: [M+H]^+^ at *m/z* 256.1166 (calcd for C_12_H_18_NO_5_, 256.1185) and [2M+Na]^+^ at *m/z* 533.2119 (calcd. for C_24_H_34_N_2_O_10_Na 533.2111). Characteristic NMR shifts indicated high similarity with the known compound subereatensin [22], however, compound **5** showed a different optical rotation value ${\left[\alpha \right]}_{D}^{21}$ −21.8 (c 0.1, CHCl_3_) compared to the reported compound$\;{\left[\alpha \right]}_{D}^{21}$ +25.51 (c 0.002, MeOH) [22]. Additionally, the absence of the NOESY correlation of the methine H-4 to the -OH group of position 3a indicated a different stereochemistry at these positions. Collectively, the relative configuration of compound **5** was deduced as 6a*R*,3a*S*,4*R*. This compound revealed a very week ECD spectrum, which could not be used for the establishment of its absolute stereochemistry. Therefore, optical rotation calculation was implemented to establish its absolute configuration. Briefly, after conformational analysis of compound **5** in chloroform by using MMFF forcefield, 13 conformers were obtained, which were subjected to geometry optimization at B3LYP/6-c31++G(d,p)/CPCM/chloroform and optical rotation at B3LYP/aug-pVTZ/CPCM/chloroform level and by considering the frequency of sodium D line for the calculation. The results demonstrated a Boltzmann-averaged specific rotation value of −59.68 (589.3 nm, sodium D line) for isomer 6a*R*,3a*S*,4*R*. Considering the sign of the calculated specific value, the absolute configuration of compound **5** could be deciphered accordingly. The deviation of the calculated numerical value and the experimental one could be possibly emanated from the conformational analysis and overestimation of the calculation methods. Therefore, compound **5** is a new natural stereoisomer of subereatensin [21]. It was identified as (6a*R*,3a*S*,4*R*)-ethyl 4-ethoxy-3a-hydroxy-2-oxo-1,2,3,3a,4,6a-hexahydrocyclopenta[b]pyrrole-6-carboxylate and given the trivial name iso-subereatensin.

Based on literature and published spectroscopic data, the other 10 isolated compounds were identified as known compounds, and specifically, they are 5-chlorocavernicolin (**6**) [21], 2-[(1r,4r)-3,5-dibromo-4-ethoxy-1-hydroxy-4-methoxycyclohexa-2,5-dien-1-yl]acetamide (**7**) [11], 2-[(1s,4s)-3,5-dibromo-4-ethoxy-1-hydroxy-4-methoxycyclohexa-2,5-dien-1-yl]acetamide (**8**) [11], N,N,N-trimethyl-3,5-dibromotyramine (**9**) [23], 2-((1s,4s)-3,5-dibromo-4-ethoxy-1-hydroxy-4-methoxycyclohexa-2,5-dien-1-yl)acetic acid (**10**) [11], 5-[3,5-dibromo-4-(2-oxo-oxazolidin-5-ylmethoxy)-phenyl]-oxazolidin-2-one (**11**) [24], aeroplysinin-2 (**12**) [11], 2,6-dibromo-4-hydroxy-4-methoxycarbonylmethylcyclohexa-2,5-dien-1-one (**13**) [11], fistularin-1 (**14**) [21], and aplysterol (**15**) [25]. Compounds **6** and **9** are reported in *Aplysina aerophoba* for the first time.

A comparison to published data also allowed the identification of the compounds isolated from *Spongia* sp. They were identified as 2-hexaprenyl-1-4-hydroquinone (**16**) [26], 2-heptaprenyl-1-4-hydroquinone (**17**) [27], 2-octaprenyl-1-4-hydroquinone (**18**) [28], 2-[24 hydroxy] octaprenyl-1-4-hydroquinone (**19**) [28], and 2-hexaprenylmethyl-2-methylchromen-6-ol (**20**) [26]. These compounds are already known constituents of *Spongia* sp.

### 3.2. Cytotoxic Properties

**Figure 6:**
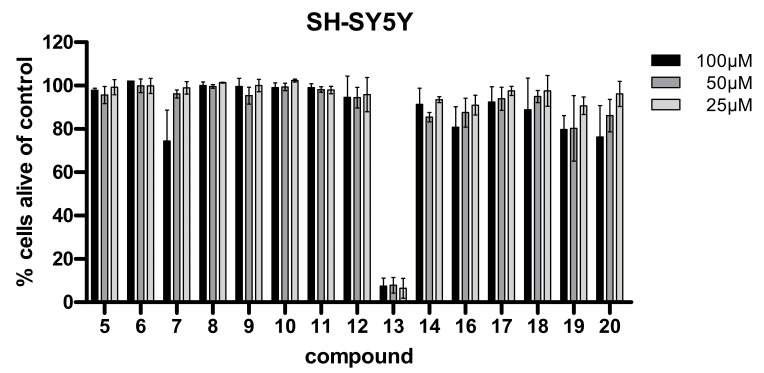
Viability of neuroblastoma cell line SH-SY5Y after treatment with different concentrations (100/50/25 µM) of the indicated compounds for 24 h.

**Figure 7:**
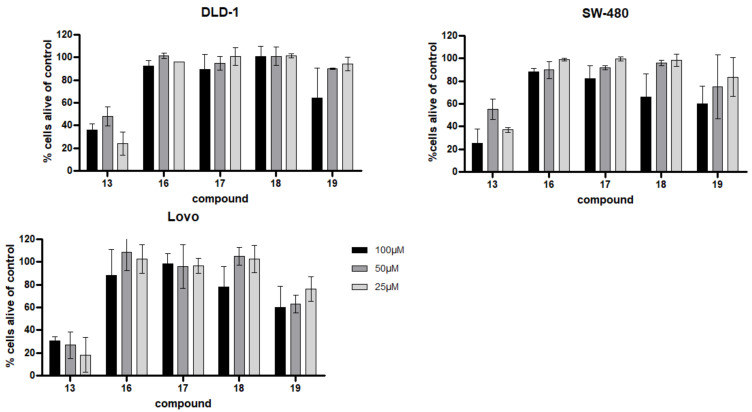
Viability of human colon adenocarcinoma cell lines DLD-1, SW-480, and Lovo treated with the respective compounds for 24 h. Viability was measured by flow cytometry (AnnV/PI staining) and % of cells alive compared to control (untreated) are shown +/− SD.

**Figure 8:**
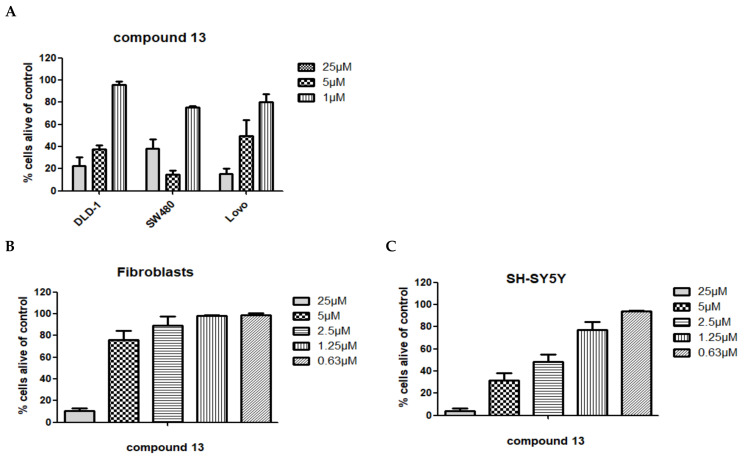
Influence of **13** at different concentrations on (**A**) different colon carcinoma cell lines, on (**B**) SH-SY5Y neuroblastoma cell line, and on (**C**) primary human fibroblasts as non-cancerous control cells. Incubation time was 24 h followed by flow cytometric analysis (AnnV/PI staining). Percentage of cells alive was calculated compared to control (no treatment).

Cytotoxic properties of almost all compounds isolated from *Spongia* sp. and *Aplysina aerophoba* to T24 bladder and AGS stomach tumor cells was determined using the common MTT assay by Mossman et al. Table 4 displays the respective, expressed as IC_50_ values, as the concentration (µM) of each test compound causing 50% effect in the respective assays. For less active compounds, the % viability at 100 µM concentration is given. Additionally, the two crude extracts were investigated (500 µg/mL). *Spongia* sp. did not show any toxicity effects whereas the *Aplysina aerophoba* extract showed a cell viability of only 25.3% after a 24 h treatment. Compounds **13**, **16**, **17**, and **19** showed strong cytotoxic effects towards AGS cells with compound **17** being the most active compound with an IC_50_ value of 0.99 µM. Compounds **12** and **18** indicated moderate cytotoxic activity towards AGS cells. However only one compound showed significant toxicity towards T24 cells, i.e., for compound **13**, an IC_50_ of 12.42 µM was determined. Compounds **7**, **14**, **17**, and **18** exerted moderate effects. In addition, the same substances (compounds **5**–**20**) were investigated for a potential apoptotic activity towards the neuroblastoma cell line SH-SY5Y in three concentrations (25 to 100 µM) using a fluorescence activated cell sorting readout (FACS). Significant cytotoxicity was observed for compound **13** in this assay (cell viability was measured below 20% for all concentrations) (Figure 6). Subsequently, the five most promising compounds according to the initial screening in the above-mentioned cell lines (compounds **13**, **16**, **17**, **18**, and **19**) were chosen for further investigations. They were additionally investigated in three colon cancer cell lines (DLD-1, SW-480 and Lovo) for potential cytotoxic effects. Again, compound **13** revealed activity in all cell lines (Figure 7), and decreased cell viability. Compound **18** and **19** showed moderate activity in the highest concentration (100 µM), with a remaining cell viability of around 60% for SW-480 and Lovo cell lines. Compound **13** was found to be cytotoxic against these colon carcinoma cell lines even at lower concentrations ranging from 1–25 µM (Figure 8A). For SH-SY5Y cells, compound **13** was titrated from 25 to 0.63 µM and the IC_50_ value was determined with 1.78 [CI 95 1.34–2.38] (Figure 8B). In comparison, etoposide, a standard cytotoxic compound used in clinical practice for neuroblastoma treatment, killed 50% of SH-SY5Y cells at a concentration of 340 nM (data not shown). We also utilized human primary fibroblasts for testing in order to delineate a general cytotoxicity of this compound. These non-cancerous cells were significantly less sensitive to **13** supporting a potential anti-cancer effect of this compound with a certain degree of selectivity (Figure 8C). (FACS Plot figures for **13** are shown in the Supplementary Materials).

**Figure 9:**
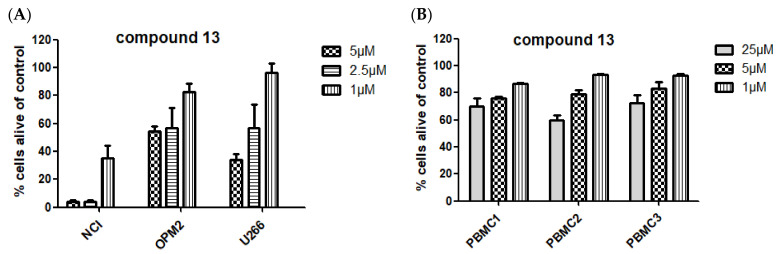
Activity of compound **13** on (**A**) hematological cancer cells (multiple myeloma cell lines) and on (**B**) primary peripheral blood mononuclear cells of three healthy donors (PBMC 1–3). Incubation time was 24 h, followed by flow cytometric analysis.

Encouraged by the positive results, compound **13** was further investigated in a hematological cancer model, i.e., multiple myeloma. Again, cytotoxicity was found at low concentrations (concentration range 1 to 25 µM), showing a decreased viability of NCI-H929, OPM-2, and U266 cell lines of 4%, 54%, and 34%, respectively, after 24 h incubation time at a concentration of 5 µM. In non-cancerous blood cells, i.e., peripheral blood mononuclear cells (PBMCs) of healthy donors, compound **13** induced significantly lower levels of cell death, again suggesting that cancer cells might be more susceptible (PBMC 1-3, Figure 9).

## 4. Discussion and Conclusions

Marine sponges are gaining the attention of the scientific community because of their unique secondary metabolites with a diversity of biological activities. Some of them are being tested in clinical trials against various diseases, mainly with the focus on anti-cancer drugs [29,30].

*A. aerophoba* and *Spongia* sp. were selected for this study because initial screening experiments already indicated that they contain interesting and possibly new metabolites.

Apart from the commonly studied bromotyrosines from *A. aerophoba* which are compounds with interesting antibacterial and antitumor activities [13,31], attention has been focused on chitin which has further applications, e.g., as scaffold in tissue engineering, pharmacological applications, and regenerative medicine [13,32]. Furthermore, the farming of *Spongia* sp. for commercial purposes as bath sponges has been going on for more than a century [33]. These sponges have a high commercial value and they are successfully employed in experimental aquaculture experiences [34]. The enormous filtering capacity of sponges has led to the suggestion that they be farmed for bioremediation purposes, e.g., to reduce the high bacterial loads resulting from sewage discharges and areas subjected to aquaculture activities [33,34]. Additionally, *Spongia* sp. have recently been studied as an alternative source of collagen which is found in their extracellular skeletal matrix [17].

In our study, twenty metabolites, mainly halogenated compounds and prenylated hydroquinones, were isolated using a purification protocol including liquid–liquid extraction, fractionation on silica gel column and Sephadex LH-20 columns, and a final clean-up step with semipreparative HPLC or flash chromatography. Four brominated metabolites and an isomer of subereatensin isolated from *A. aerophoba* have been identified as novel natural products and two additional brominated compounds were reported from *A. aerophoba* for the first time.

It is already known that some of the isolated compounds exert cytotoxic and antimicrobial properties, e.g., aeroplysinin-2 is known to possess moderate antibacterial activity against the Gram-positive bacteria *Staphylococcus lentus*, *Propionibacterium acnes*, and *Bacillus subtilis* [31], the mouse fibroblasts NIH-3T3, the human hepatocellular carcinoma cell line HepG2, and the human colon adenocarcinoma cell line HT-29 [31]. Additionally, aeroplysinin-1 shows cytotoxic effects against human neuroblastoma (SH-SY5Y) [13]. Further, 2-Octaprenyl-1-4-hydroquinone has been reported to inhibit marine bacterial strains (both Gram-positive and -negative bacteria) [35], while 2-[24 hydroxy] octaprenyl-1-4-hydroquinone revealed moderate cytotoxic activity against C98 cells and good antibacterial activity against *S. aureus* and *E. cloacae* [36]. In addition, 2,6-Dbromo-4-hydroxy-4-methoxycarbonylmethylcyclohexa-2,5-dien-1-one, the most cytotoxic compound in our study, had only been investigated for its anti-microbial properties showing weak effects [37].

When evaluating the possible cytotoxic effects of the isolated metabolites, only a few of them showed activities in the investigated cell lines. For AGS stomach tumor cells, compound **17** was found to be the most toxic, while compound **13** showed the highest cytotoxic activity in all other cell lines tested. Therefore, this compound seems to be interesting for further studies, especially because our data indicated selectivity towards cancer cells.

Sponges often contain diverse and abundant microbial communities in their tissues and in many cases, the associated bacterial communities account for over 40% of the biomass of their hosts [30,34]. In the past few years, more and more evidence has accumulated in which a part of the isolated metabolites are not produced by the sponges themselves, but instead, they are products of the metabolic activities of bacteria living in the sponge tissue [34]. This also could be the case for the compounds isolated within this study. Our results suggest that *A. aerophoba* and *Spongia* sp., among other demosponges which currently attract increased interest in the scientific community, are a rich source of interesting compounds. This refers to unique chemical structures as well as promising bioactivities.

## Figures and Tables

**Table 1:** ^1^H NMR Data (*δ* in ppm, *J* in Hz) of compounds **1** (400 MHz) and **4** (600 MHz).

No.	Compound 1 (400 MHz)		Compound 4 (600 MHz)	
	*δ*_H_ (*J* in Hz)	*δ*_C_	*δ*_H_ (*J* in Hz)	*δ*_C_
1		159.3		153.9
2	3.38 t (7.2)	39.2	6.76 d (8.4)	117.1
3	2.02 m	31.4	7.07 dd (1.8/8.4)	130.5
4	4.02 t (6.4)	72.2		130.8
5		152.9	7.37 d (1.8)	134.7
6		119.0		110.6
7	7.49 s	134.5	3.78 s	28.8
8		140.8		153.1
9	7.49 s	134.5		165.8
10		119.0	3.48 t (6.6)	36.7
11	2.80 t (7.6)	35.9	2.48 t (6.6)	35.6
12	3.19 t (7.6)	46.2		176.9 *
13	4.07 q (7.2)	61.8		
14	1.23 t (7.2)	15.2		

Measured in MeOD, * *δ***_C_** established from the HMBC spectrum.

**Table 2:** ^1^H NMR Data (*δ* in ppm, *J* in Hz) of compounds **2** (400 MHz) and **3** (600 MHz).

No.	Compound 2 ^a^		Compound 3 ^b^	
	*δ*_H_ (*J* in Hz)	*δ*_C_	*δ*_H_ (*J* in Hz)	*δ*_C_
1		154.0		153.0
2, 6		118.8–119.2 **		118.1
3, 5	7.49 s	134.7	7.48 brs	133.7
4		137.5		136.4 *
7	3.85 s	28.9	3.89 s	39.0
8		152.2		152.6 *
9		165.5		163.5
10	3.55 t (6.8)	38.0	3.54 m 3.76 m	42.3
11	2.06 m	30.6	4.21 m	69.9
12	4.01 t (6.0)	72.4	4.00 dd (4.8,9.0) 4.09 dd (4.8,9.0)	74.6
13		153.0		152.9
14, 18		118.8, 119.2 *		118.9
15, 17	7.48 s	134.5	7.53 brs	130.2
16		140.0		137.8 *
19	2.84 t (7.6)	35.0	5.54 t (8.0)	75.8
20	3.25 t (7.6)	46.4	3.50 t (8.0) 4.00 t (8.0)	48.1
21	3.81 s	61.2		158.5
22			3.83 s	61.0
NH			7.22 m	

^a^ Measured in MeOD, ^b^ Measured in chloroform-*d* * *δ***_C_** established from the HMBC spectrum, ** Overlapping signals, Measured in MeOD.

**Table 3:** ^1^H NMR Data (*δ* in ppm, *J* in Hz) of compound **5** (600 MHz).

No.	*δ*_H_ (*J* in Hz)	*δ*_C_	NOESY	COSY	HMBC
2		174.3			
3′	2.70 d (17.4)	43.1		H-3′′	C-2,C-3a,C-4,C-6a
3′′	2.48 d (17.4)	43.1	H-4	H-3′	C-2,C-4,C-6a
3a		81.2			
4	4.20 m	84.4	H-3′′, H-5, H-9, H-10	H-5	C-3,5,6,10
5	6.83 d (1.8)	140.1		H-4	C-3a,C-4,C-6a,C-7
6		139.5			
6a	4.48 brs	68.6	OH		C-2,C-3a,C-5
7		163.7			
8		61.6	H-9	H-9	C-7,C-9
9	1.31 t (7.2)	14.4	H-8, H-4	H-8	C-8
10	3.73 m	67.5	H-5, H-11	H-10	C-4,C-11
11	1.28 t (7.2)	15.6	H-10a	H-11	C-10
OH	6.06 brs		H-6a		C-3,3a,6a

Measured in chloroform-*d.*

**Table 4:** Summary of all compounds and their IC_50_ values of cell viability/% viability at 100 µM. (95% confidence intervals in parentheses).

Compound	AGS	T24
	IC_50_ µM (CI 95 ±)	IC_50_ µM (CI 95 ±)
**5**	Not active	Not active
**6**	Not active	Not active
**7**	Not active	54.57% at 100 µM (21.88–107.50)
**8**	Not active	Not active
**9**	Not active	Not active
**10**	Not active	Not active
**11** *	-	-
**12**	54.57% at 100 µM (17.87–86.17)	Not active
**13**	10.14 (8.03 to 12.82)	12.42 (10.65 to 14.48)
**14**	Not active	56.20% at 100 µM (16.81–70.79)
**15** *	-	-
**16**	5.33 (4.03 to 7.06)	Not active
**17**	0.994 (0.61 to 1.61)	66.63% at 100 µM (45.10–88.16)
**18**	33.41% at 100 µM (13.02–53.82)	34.38% at 100 µM (7.454–76.22)
**19**	8.09 (5.68 to 11.52)	Not active
**20**	Not active	Not active
*Spongia* sp. extract 500 µg /mL	Not active	Not active
*A. aerophoba* extract 500 µg/mL	25.26%	Not active

* not tested because of poor solubility.

## Data Availability

Not applicable.

## Supplementary Materials

The following are available online at https://www.mdpi.com/article/10.3390/biom11050723/s1, HRMS, 1D and 2D NMR, IR spectra of all the new compounds **1**–**5**, HPLC separation of the *Spongia* spp. extract, conformers of compound **5** and their Boltzmann averaging along with their calculated specific rotation values.
