# Supplementary material for: Cytotoxic Compounds of Two Demosponges (Aplysina aerophoba and Spongia sp.) from the Aegean Sea
Source: Biomolecules. 2021 May 12;11(5):723. doi: 10.3390/biom11050723 (PMC8151441; doi:10.3390/biom11050723)
Supplement: Supplementary file 1 [file biomolecules-11-00723-s001.zip › biomolecules-1158856-supplementary.pdf]

Supplementary materials

Cytotoxic compounds of two demosponges (*Aplysina aerophoba* and *Spongia sp.*) from the Aegean Sea

Maria Orfanoudaki[1], Anja Hartmann[1,*], Mostafa Alilou[1], Naida Mehic[1], Marcel Kwiatkowski [2], Karin Jöhrer[3], Hieu Nguyen Ngoc [4,5], Andreas Hensel[6], Richard Greil[3,7] and Markus Ganzera[1]

[1] Institute of Pharmacy, Pharmacognosy, University of Innsbruck, Innrain 80-82, Innsbruck 6020, Austria; orfmaria@gmail.com (M.O.); Anja.Hartmann@uibk.ac.at (A.H.); Naida.Mehic@student.uibk.ac.at (N.M.); mostafa.alilou@uibk.ac.at (M.A.); hieu.nguyenngoc@phenikaa-uni.edu.vn (H.N.N.); markus.ganzera@uibk.ac.at (M.G.)

[2] Functional Proteo-Metabolomics, Department of Biochemistry, University of Innsbruck, Innrain 80-82, 6020 Innsbruck, Austria Marcel.Kwiatkowski@uibk.ac.at (M. K.);

[3] Tyrolean Cancer Research Institute, Innsbruck, Austria; karin.joehrer@tkfi.at (K. J.)

[4] Faculty of Pharmacy, PHENIKAA University, Hanoi 12116, Vietnam; hieu.nguyenngoc@phenikaa-uni.edu.vn (H.N.N.)

[5] PHENIKAA Research and Technology Institute (PRATI), A&A Green Phoenix Group JSC, No. 167 Hoang Ngan, Trung Hoa, Cau Giay, Hanoi 11313, Vietnam

[6] Institute of Pharmaceutical Biology and Phytochemistry, University of Münster, Corrensstraße 48, D-48149 Münster, Germany, ahensel@uni-muenster.de (A.HE.)

[7] IIIrd Medical Department, Paracelsus Medical University Salzburg, Müllner Hauptstraße 48, Salzburg 5020, Austria, Cancer Cluster Salzburg, Salzburg, Austria (r.greil@salk.at) (R.G.)

***** Correspondence: Anja.Hartmann@uibk.ac.at; Tel.: +43 512 507-58430

Contents

Figure S1. ^{1}H NMR spectrum of compound 1 in MeOH....4

Figure S2. COSY spectrum of compound 1 in MeOH....4

Figure S3. HSQC spectrum of compound 1 in MeOH....5

Figure S4. HMBC spectrum of compound 1 in MeOH....5

Figure S5. ^{13}C NMR spectrum of compound 1 in MeOH....6

Figure S6. High-resolution mass spectrum of compound 1....6

Figure S7. MS/MS fragmentation and suggested fragmentation pattern of compound 1....7

Figure S8. IR spectrum of compound 1....7

Figure S9. 1H NMR spectrum of compound 2 in MeOH....8

Figure S10. COSY spectrum of compound 2 in MeOH....9

Figure S11. HSQC spectrum of compound 2 in MeOH....9

Figure S12. HMBC spectrum of compound 2 in MeOH....9

Figure S13. 13C NMR spectrum of compound 2 in MeOH....10

Figure S14. High-resolution mass spectrum of compound 2....10

Figure S15 MS/MS fragmentation and suggested fragmentation pattern of compound 2....11

Figure S16. IR spectrum of compound 2....11

Figure S17. 1H NMR spectrum of compound 3 in CDCl3....12

Figure S18. COSY spectrum of compound 3 in CDCl3....12

Figure S19. HSQC spectrum of compound 3 in CDCl3....13

Figure S20. HMBC spectrum of compound 3 in CDCl3....13

Figure S21. 13C NMR spectrum of compound 3 in CDCl3....14

Figure S22. NOESY spectrum of compound 3 in CDCl3....14

Figure S23. High-resolution mass spectrum of compound 3....15

Figure S24 MS/MS fragmentation and suggested fragmentation pattern of compound 3....15

Figure S25. IR spectrum of compound 3....16

Figure S26. ^{1}H NMR spectrum of compound 4 in MeOH....16

Figure S27. COSY spectrum of compound 4 in MeOH....17

Figure S28. HSQC spectrum of compound 4 in MeOH....17

Figure S29. HMBC spectrum of compound 4 in MeOH....18

Figure S30. ^{13}C NMR spectrum of compound 4 in MeOH....18

Figure S31. High-resolution mass spectrum of compound 4....19

Figure S32. IR spectrum of compound 4....19

Figure S33. ^{1}H NMR spectrum of compound 5 in $CHCl_3$....20

Figure S34. COSY spectrum of compound 5 in $CHCl_3$....20

Figure S35. HSQC spectrum of compound 5 in $CHCl_3$....21

Figure S36. HMBC spectrum of compound 5 in $CHCl_3$..21

Figure S37. ^{13}C NMR spectrum of compound 5 in $CHCl_3$..22

Figure S38. NOESY spectrum of compound 5 in $CHCl_3$..22

Figure S39. High-resolution mass spectrum of compound 5..23

Figure S40. HPLC separation of the *Spongia* spp. extract..23

Figure S41. The most stable conformers (population > 3%) of compound 5 optimized at B3LYP/6-31G++(d,p)/CPCM/chloroform level..24

Figure S 42: SH-SY5Y cells and fibroblasts (FB), respectively, were treated with different concentrations of compound 13 (25 µM – 0.63µM) or left untreated (control). A representative analysis is shown. Cells alive are displayed in the lower left quadrant (Q3), early apoptotic cells are shown in Q4 (lower right), late apoptotic cells appear in the upper right quadrant (Q2) and necrotic cells would appear in the upper left quadrant (Q1)..25

Figure S 43: SW480 colon carcinoma cells were treated with different concentrations of compound 13 (100 µM – 25 µM) or left untreated (control). A representative analysis is shown in the upper row. In the lower row a representative experiment using different concentrations (25 µM-1 µM and untreated control) is displayed. Cells alive are displayed in the lower left quadrant (Q3), early apoptotic cells are shown in Q4 (lower right), late apoptotic cells appear in the upper right quadrant (Q2) and necrotic cells would appear in the upper left quadrant (Q1)..25

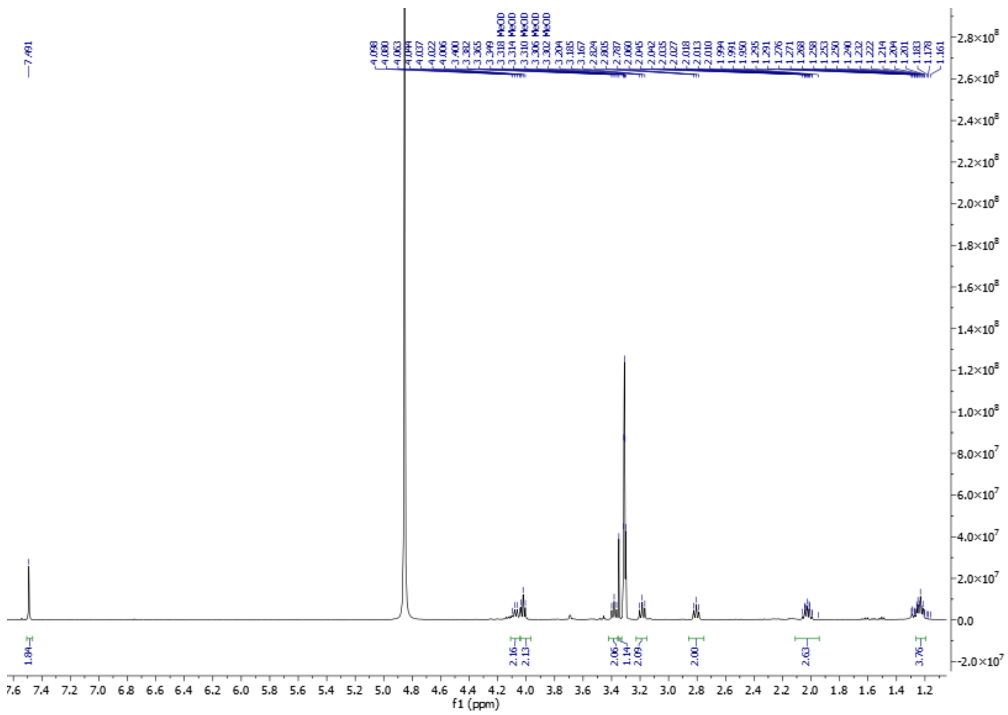

Figure S1. ^{1}H NMR spectrum of compound **1** in MeOH

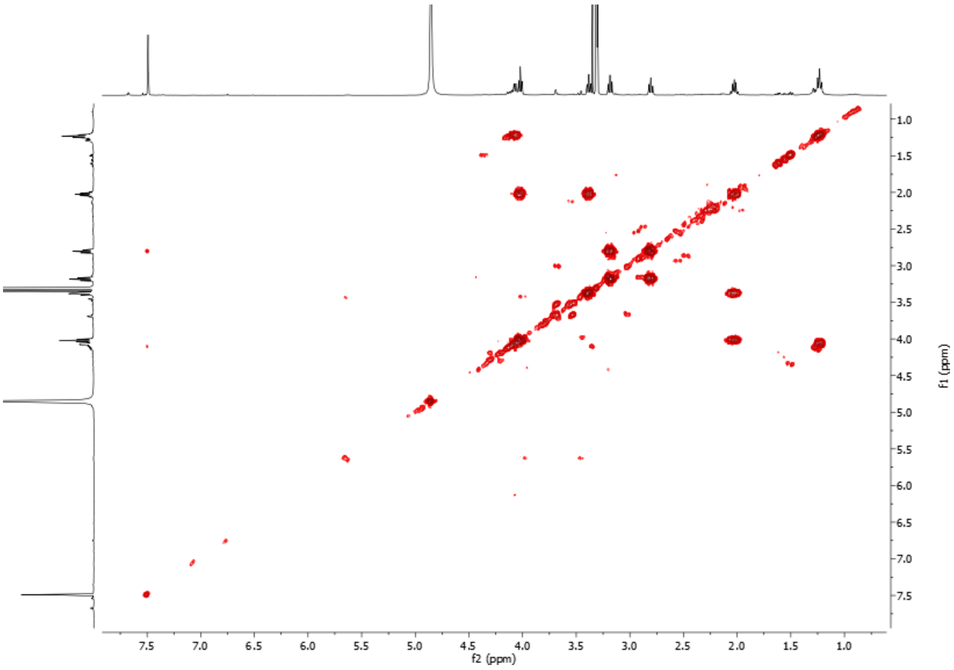

Figure S2. COSY spectrum of compound **1** in MeOH

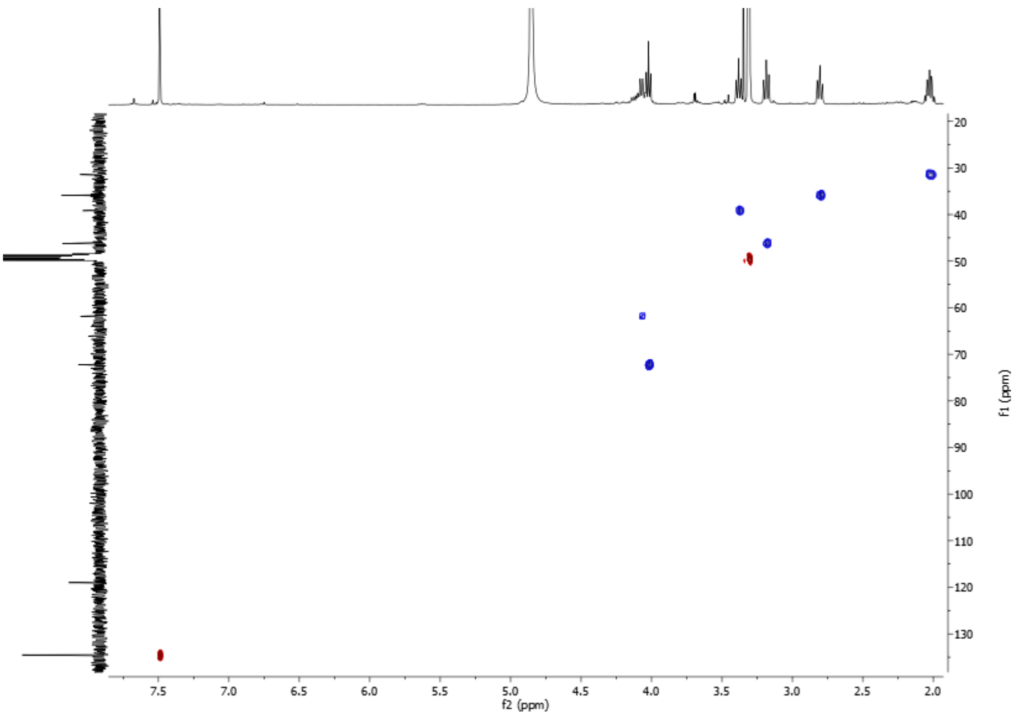

Figure S3. HSQC spectrum of compound **1** in MeOH

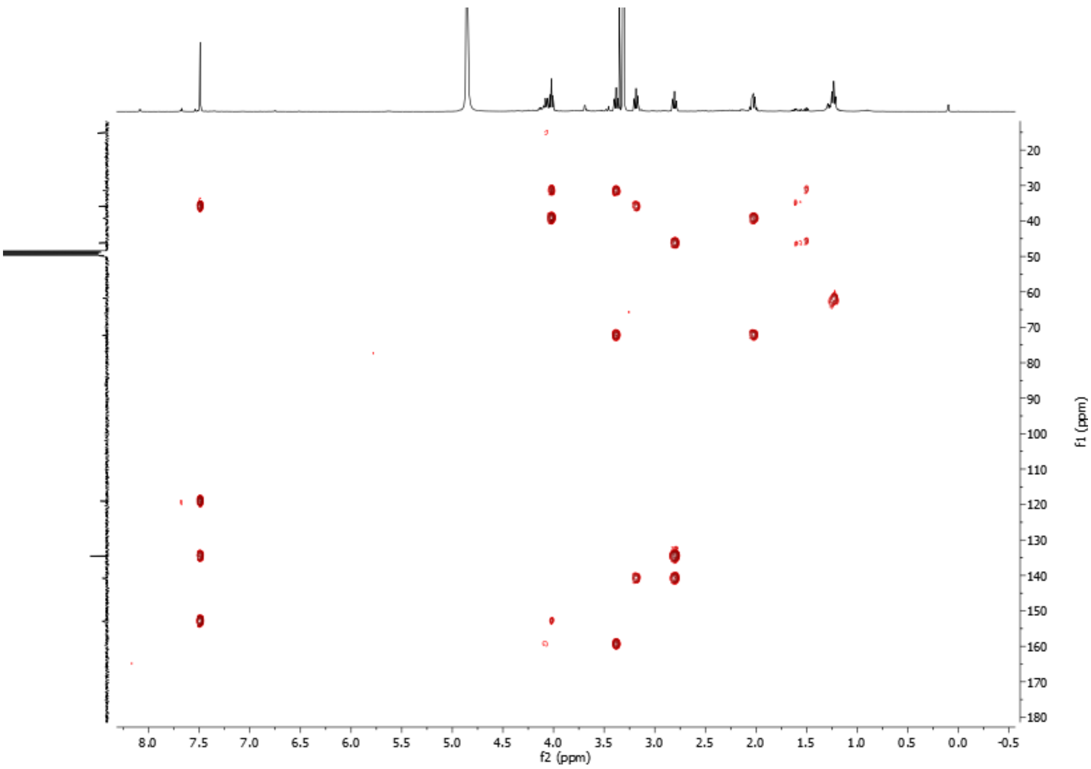

Figure S4. HMBC spectrum of compound **1** in MeOH

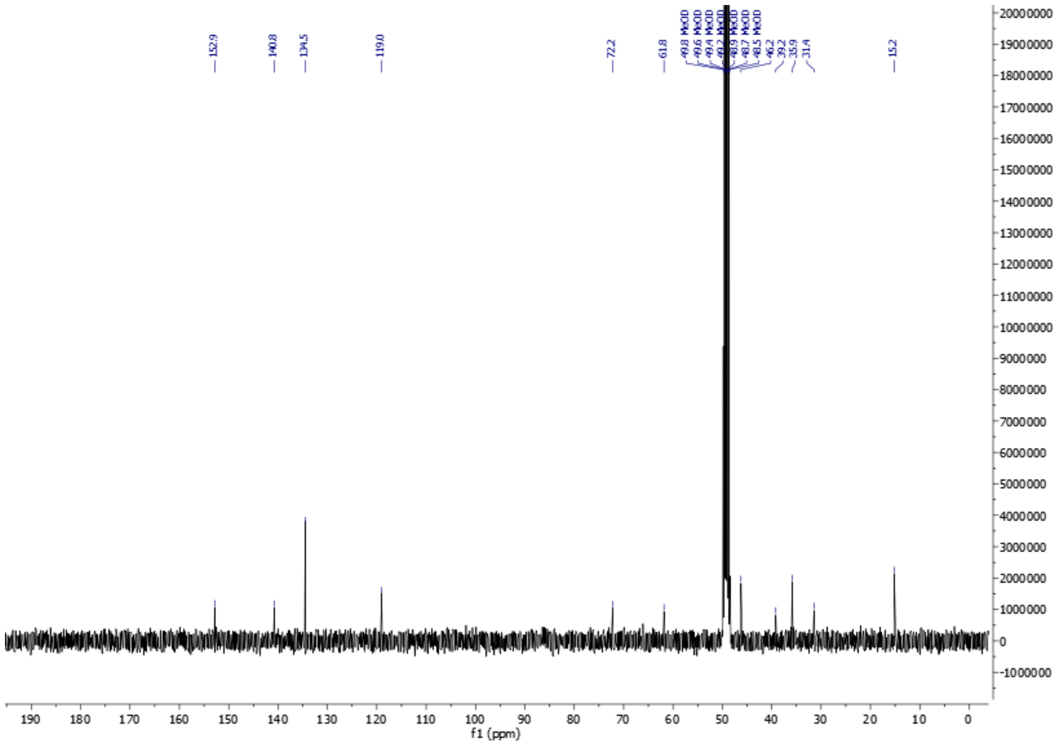

Figure S5. ^{13}C NMR spectrum of compound **1** in MeOH

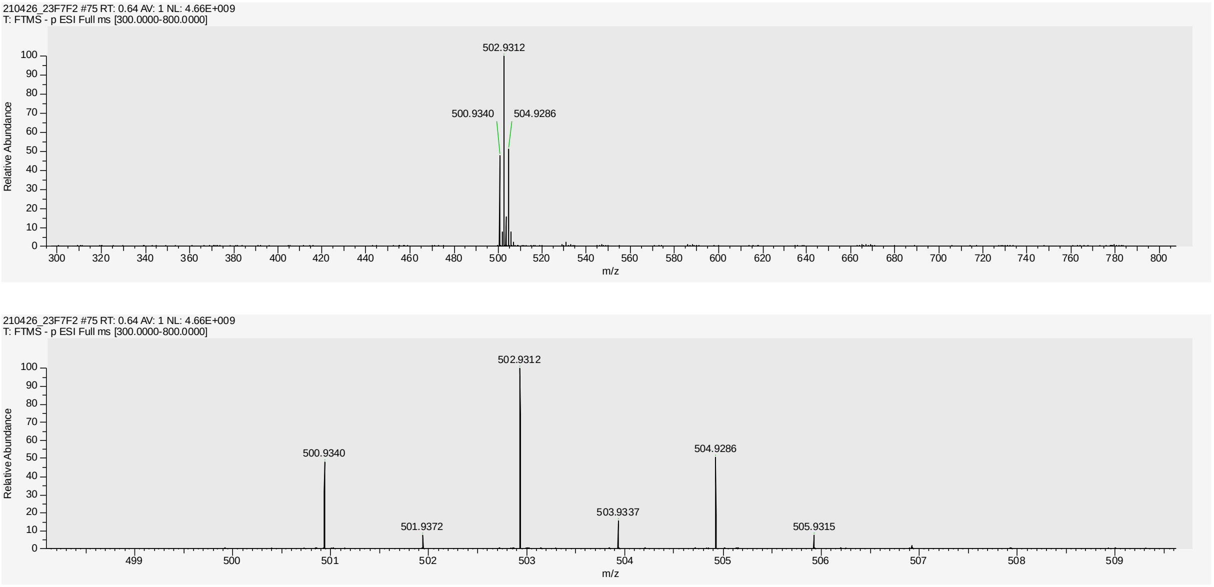

Figure S6. High-resolution mass spectrum of compound **1**

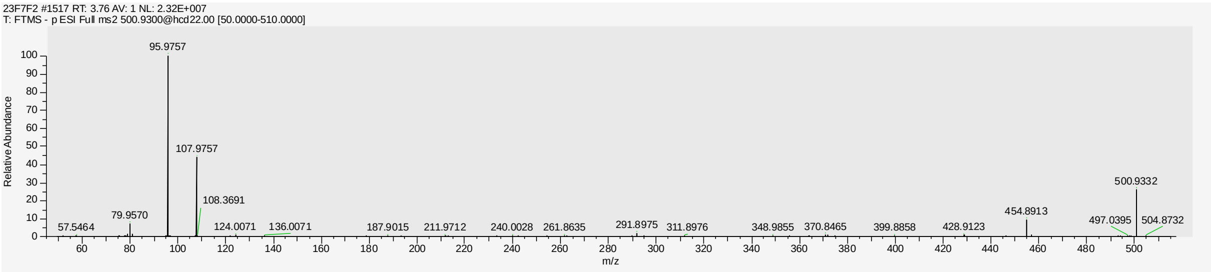

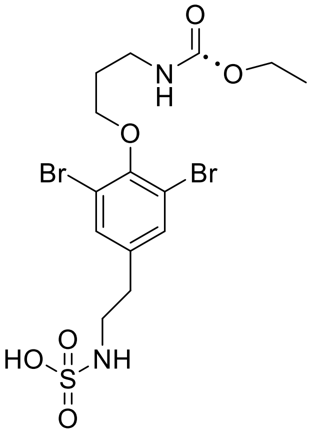

Chemical Formula: $C_{12}H_{15}Br_2N_2O_5S^{\bullet}$
Exact Mass: 456.9068

Figure S7. MS/MS fragmentation and suggested fragmentation pattern of compound **1**; Normalized HCD energy: 22%, precursor ion: 500.9332

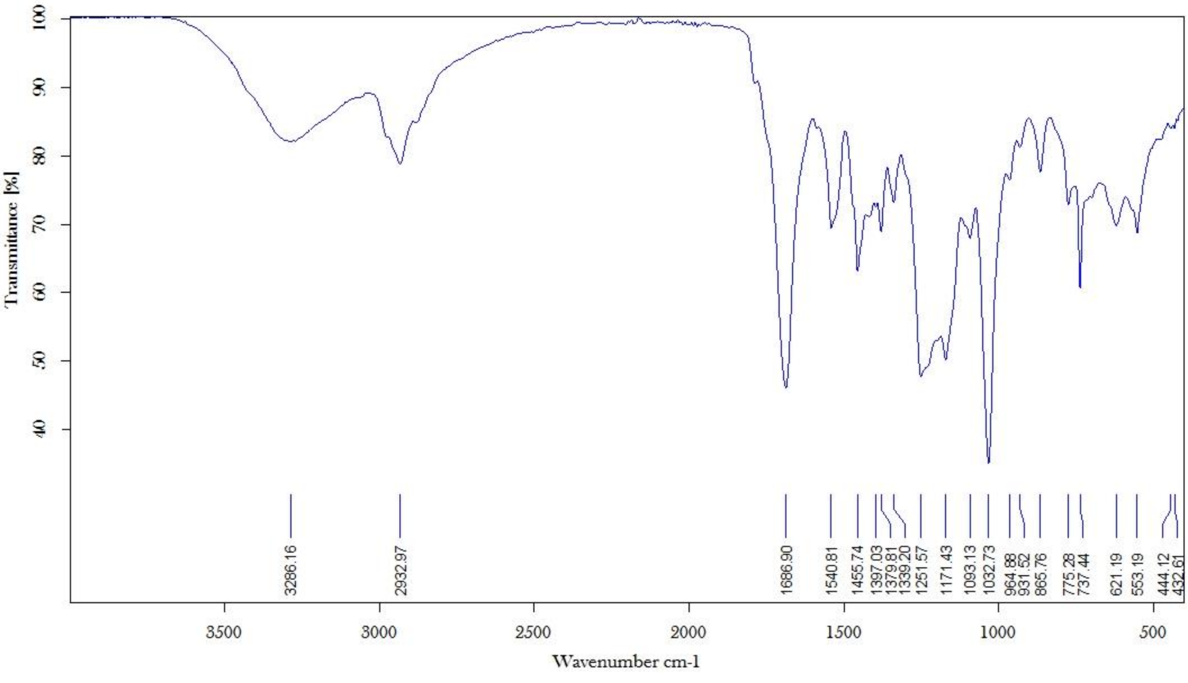

Figure S8. IR spectrum of compound **1**

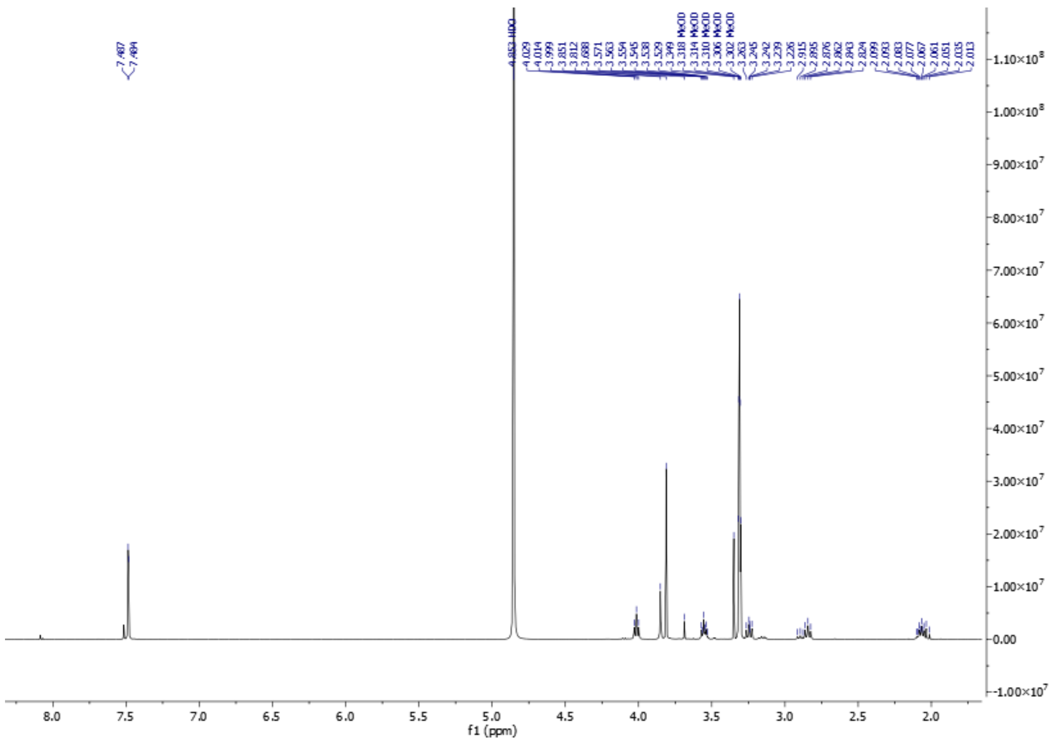

Figure S9. ^{1}H NMR spectrum of compound **2** in MeOH

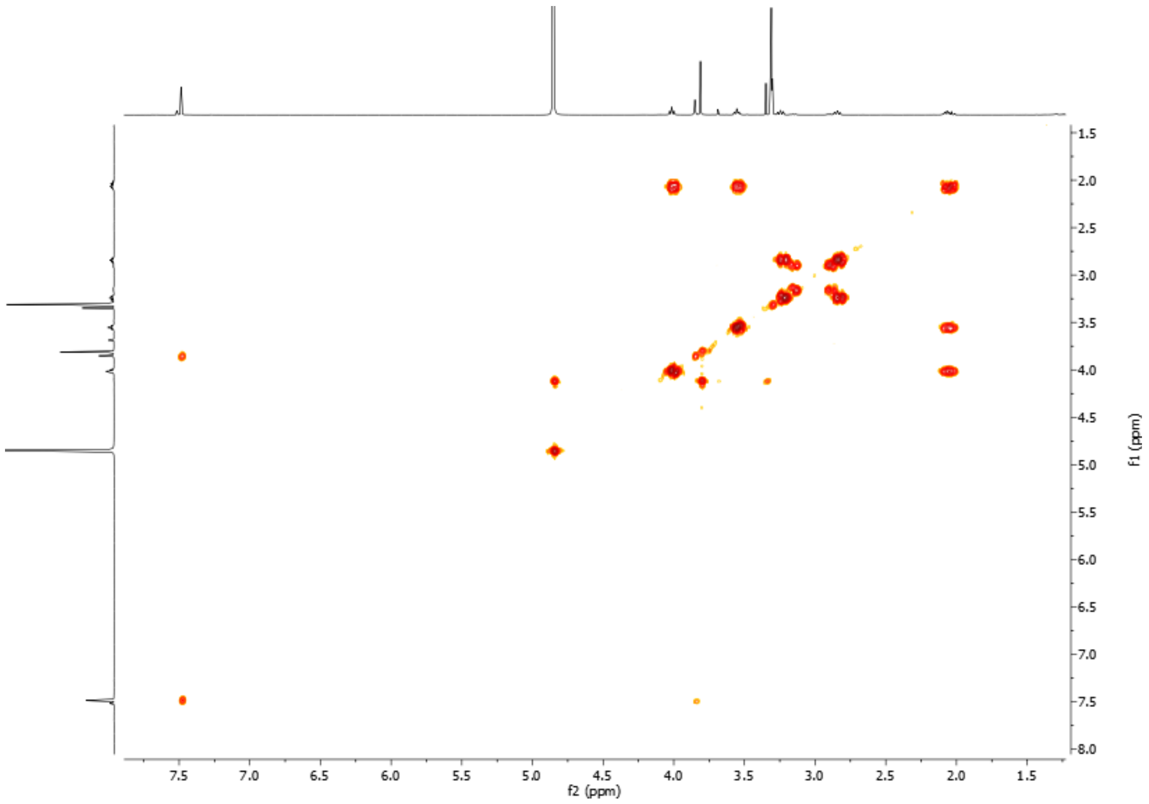

Figure S10. COSY spectrum of compound **2** in MeOH

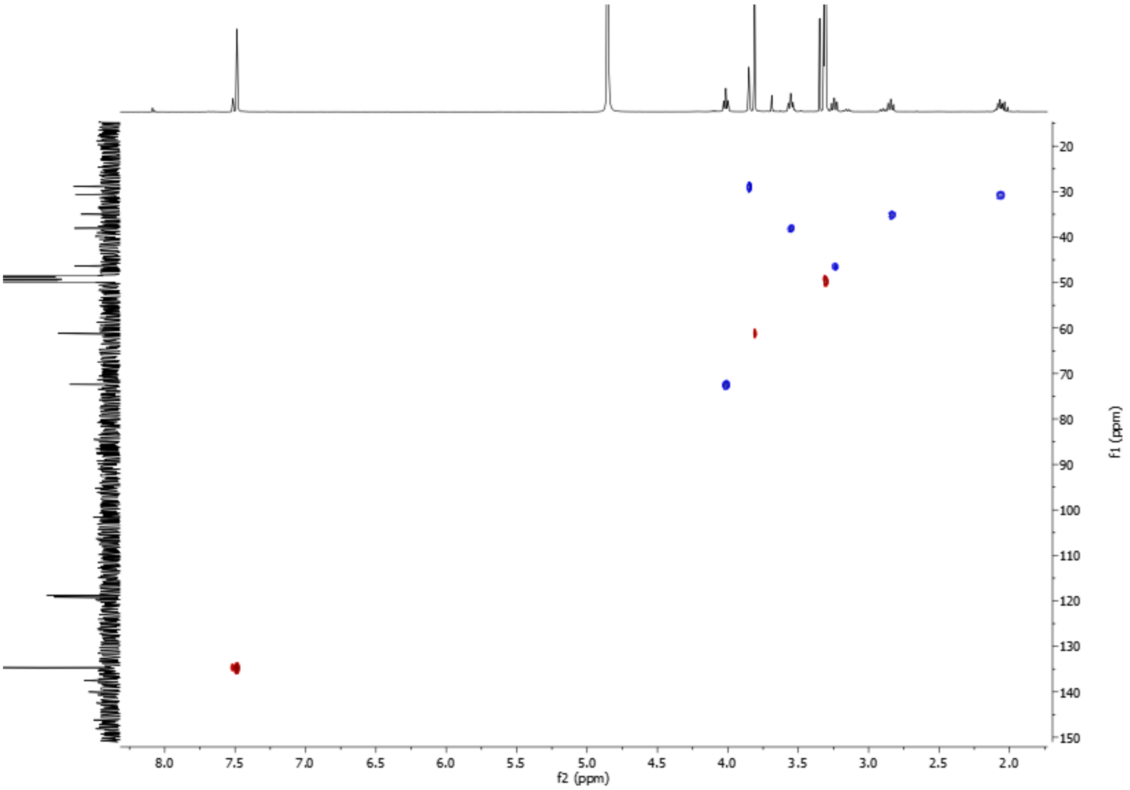

Figure S11. HSQC spectrum of compound **2** in MeOH

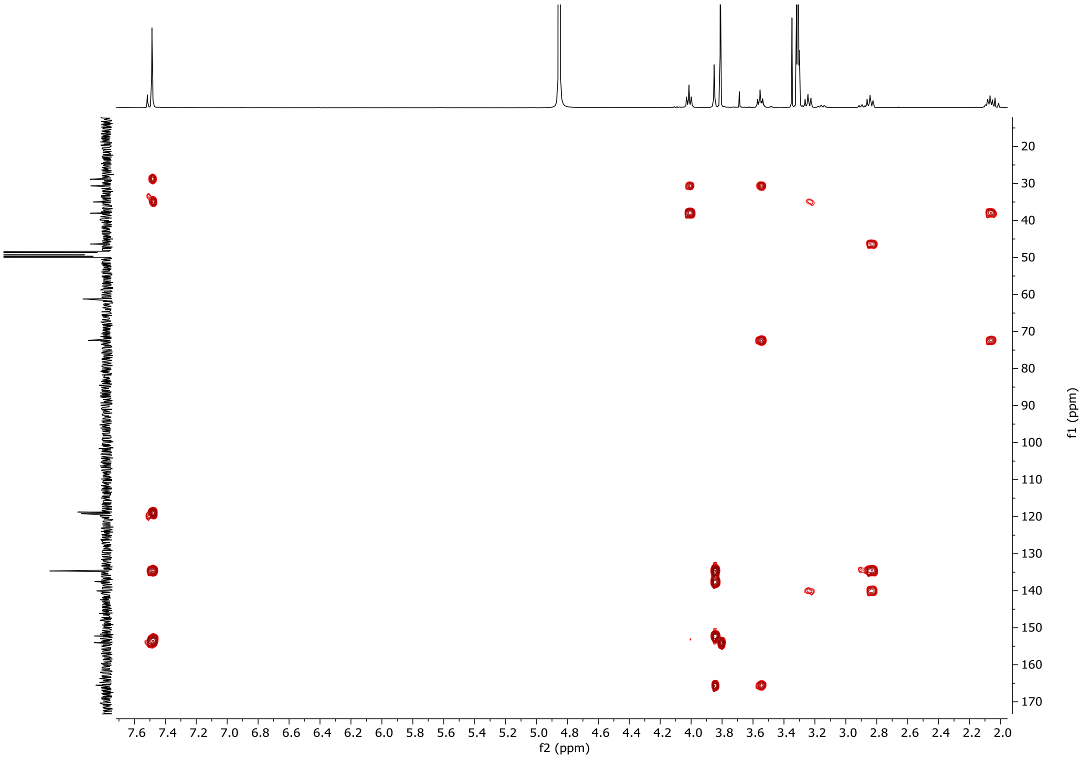

Figure S12. HMBC spectrum of compound **2** in MeOH

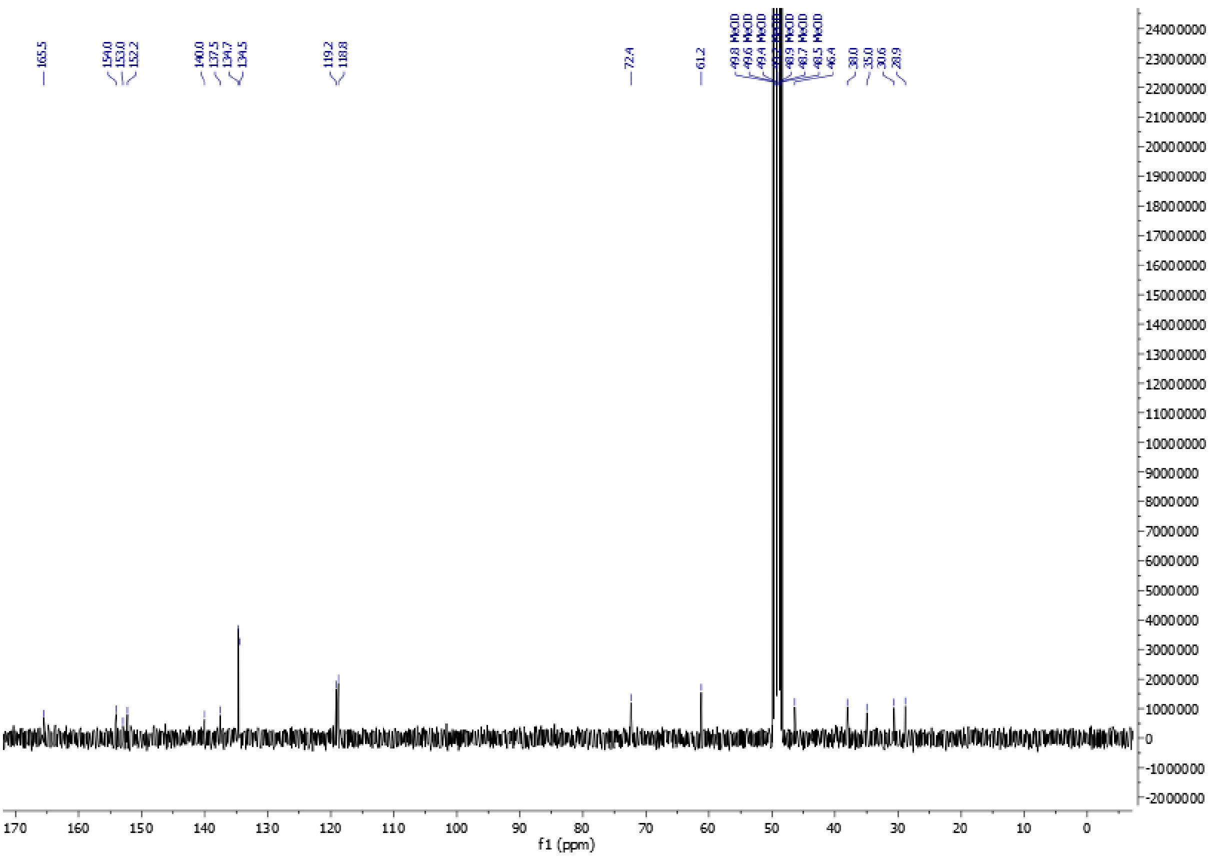

Figure S13. ^{13}C NMR spectrum of compound **2** in MeOH

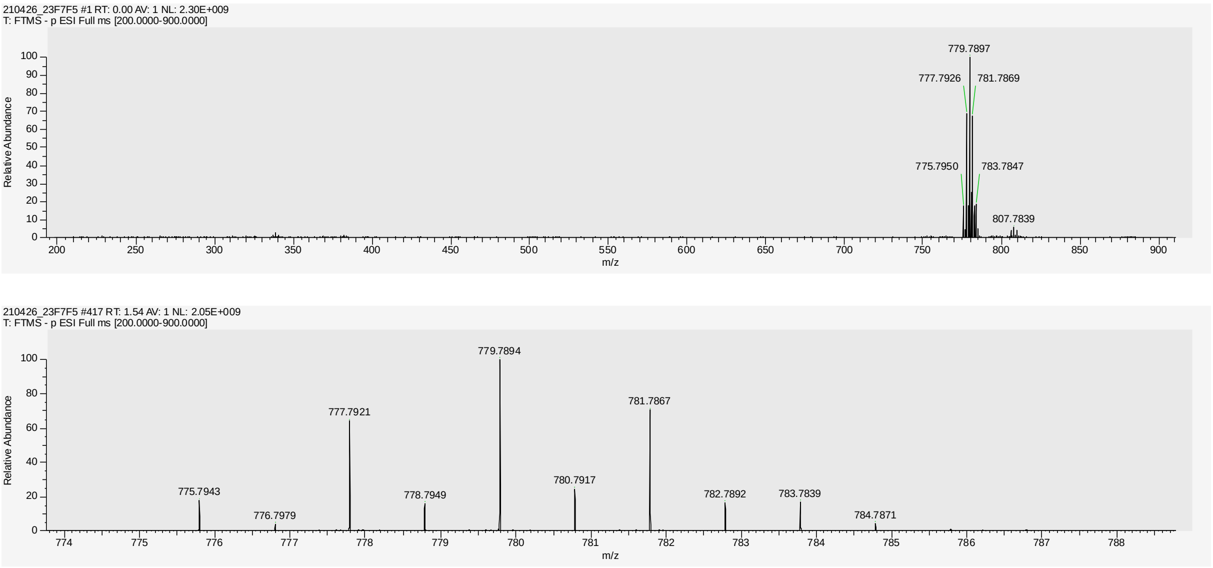

Figure S14. High-resolution mass spectrum of compound **2**

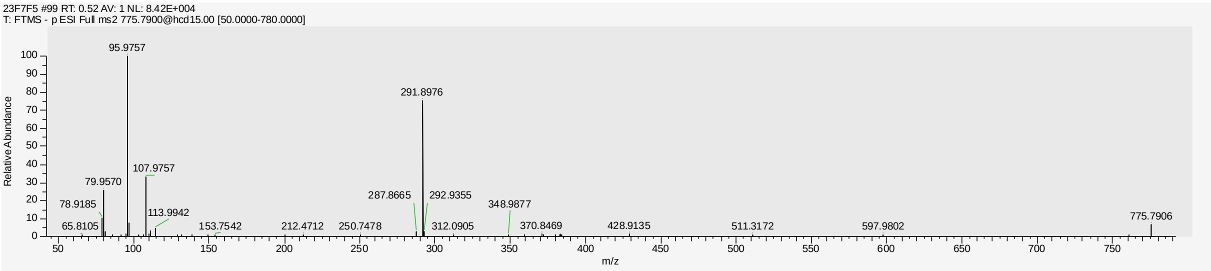

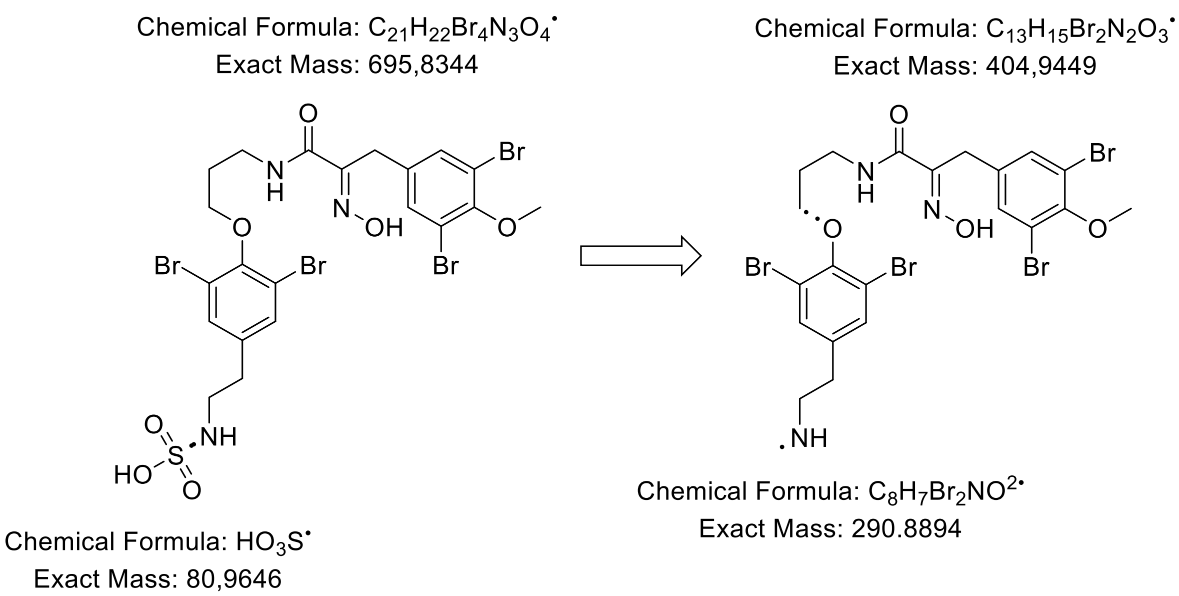

Figure S15 MS/MS fragmentation and suggested fragmentation pattern of compound **2**; Normalized HCD energy: 15%, precursor ion: 775,7906

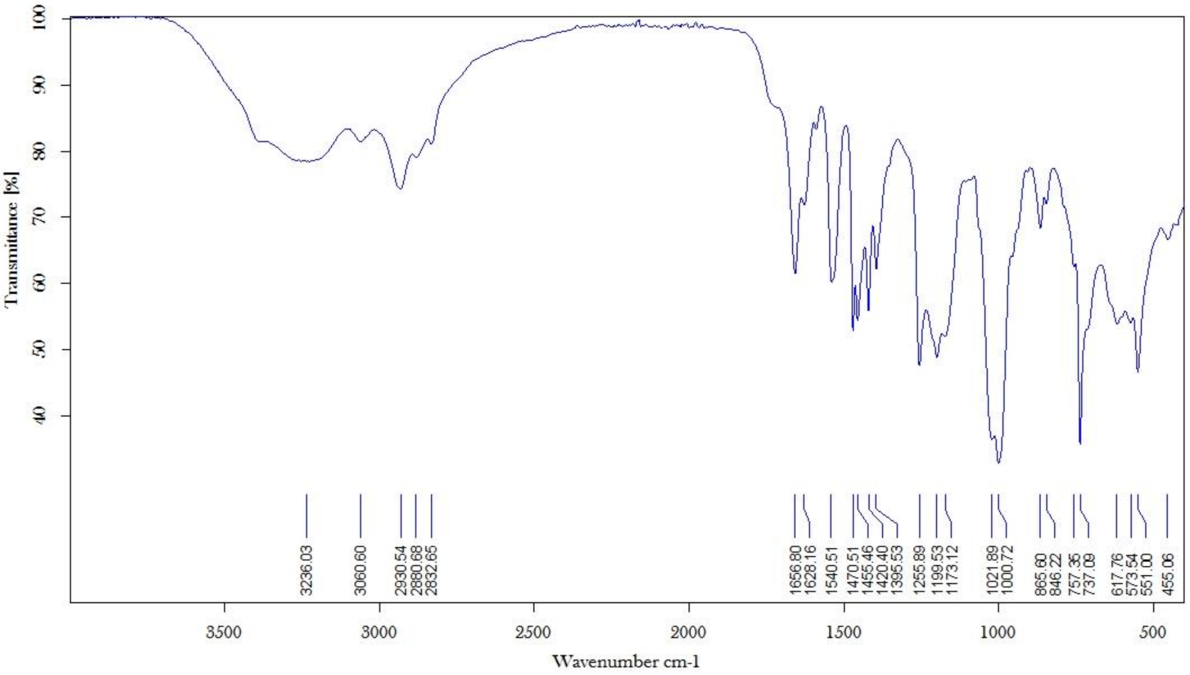

Figure S16. IR spectrum of compound **2**

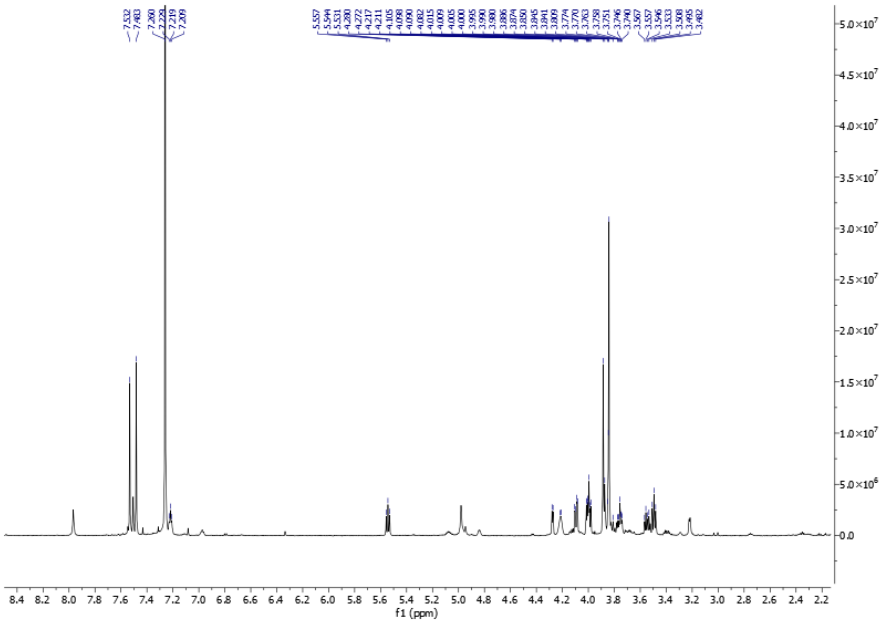

Figure S17. ^{1}H NMR spectrum of compound **3** in $CDCl_3$

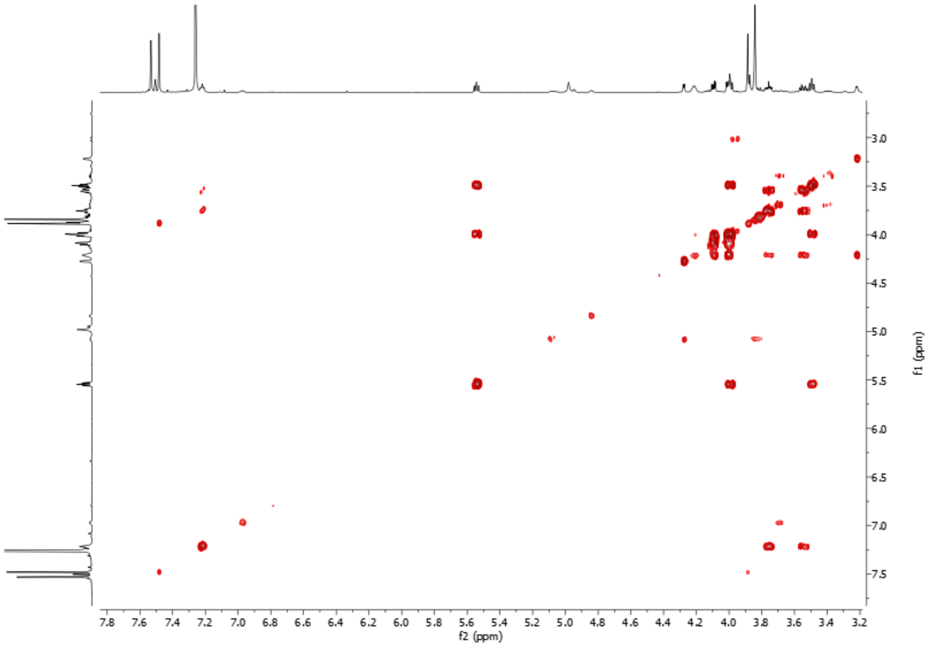

Figure S18. COSY spectrum of compound **3** in $CDCl_3$

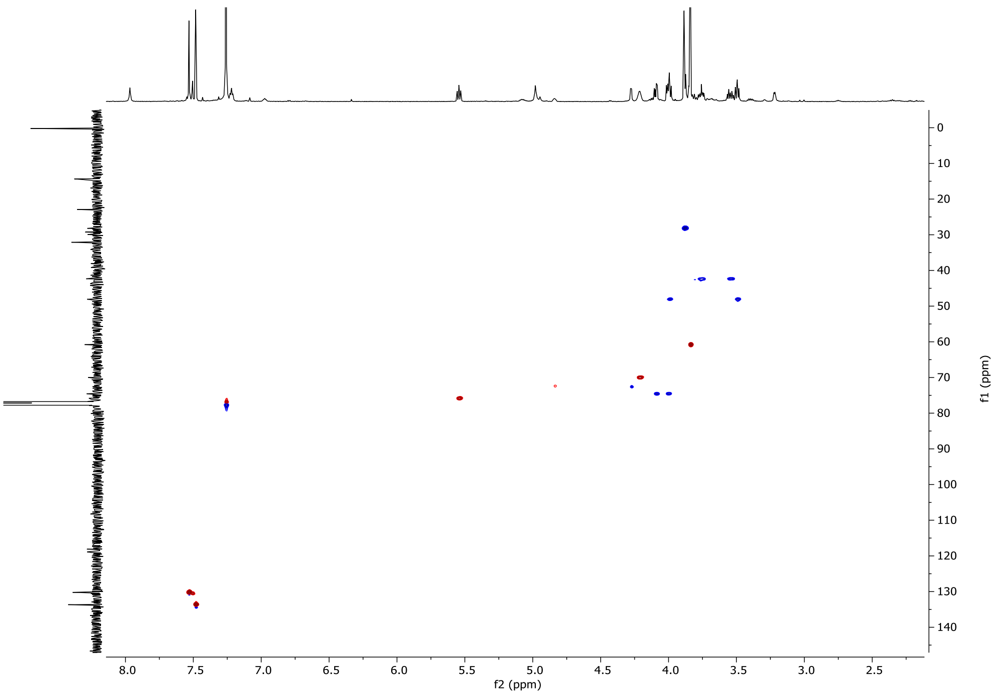

Figure S19. HSQC spectrum of compound **3** in $CDCl_3$

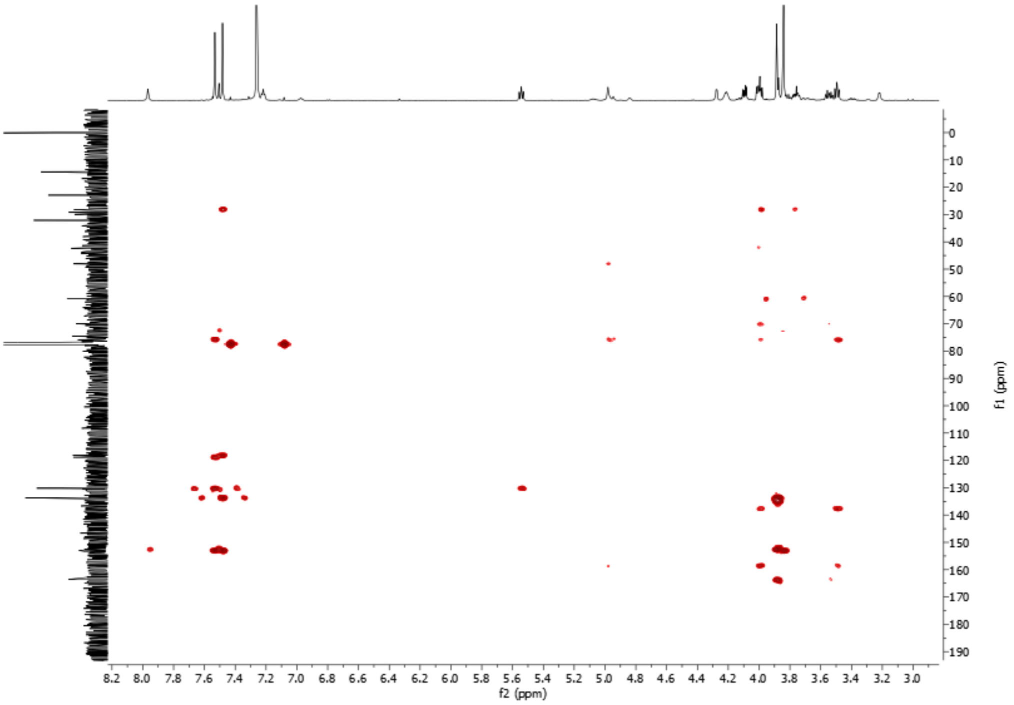

Figure S20. HMBC spectrum of compound **3** in $CDCl_3$

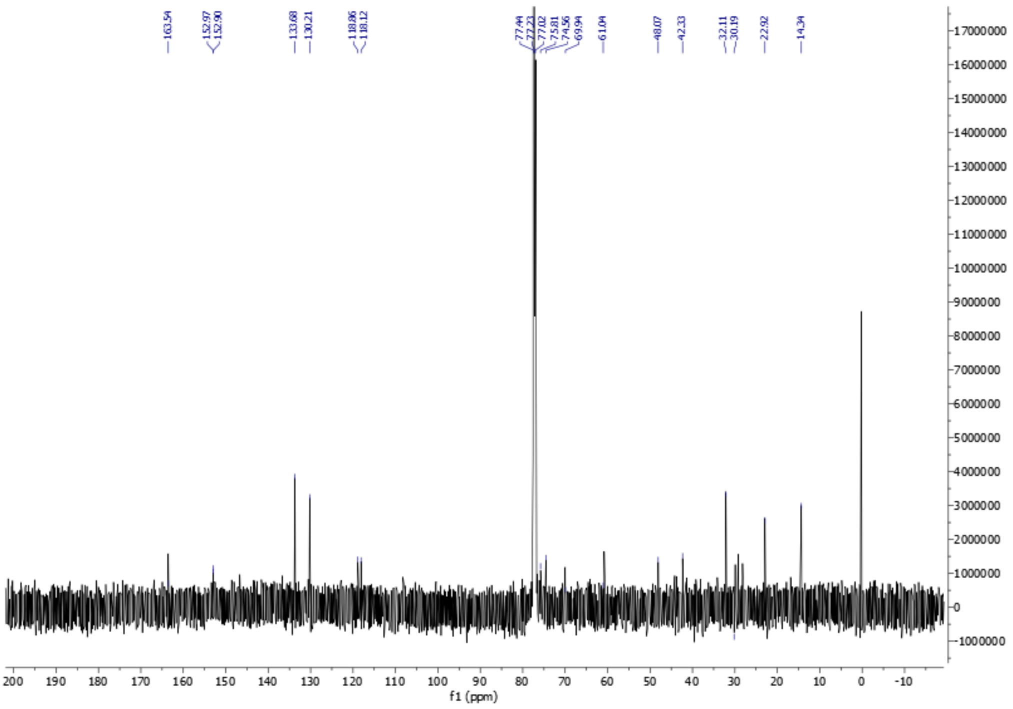

Figure S21. ^{13}C NMR spectrum of compound **3** in $CDCl_3$

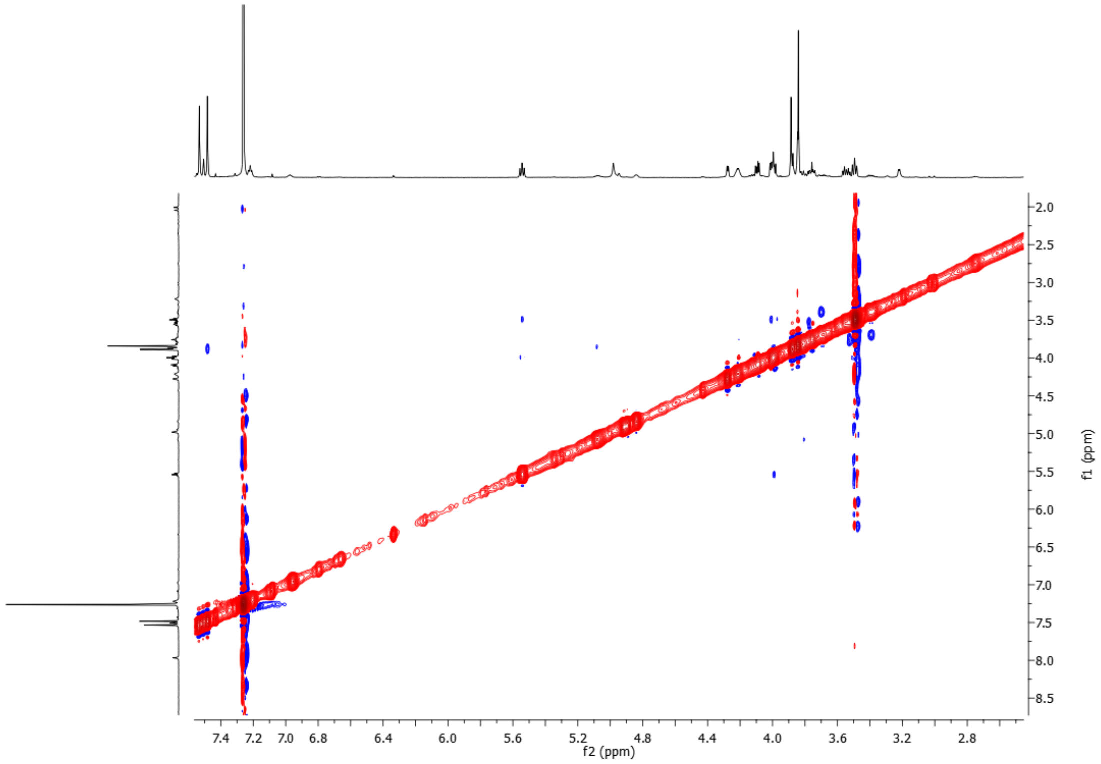

Figure S22. NOESY spectrum of compound **3** in $CDCl_3$

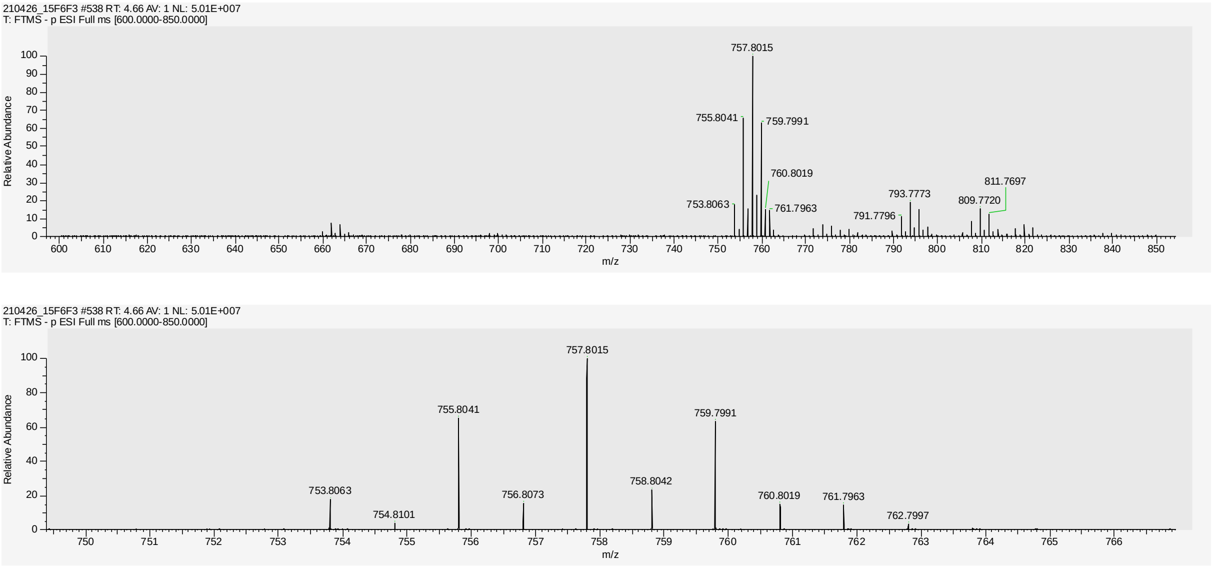

Figure S23. High-resolution mass spectrum of compound **3**

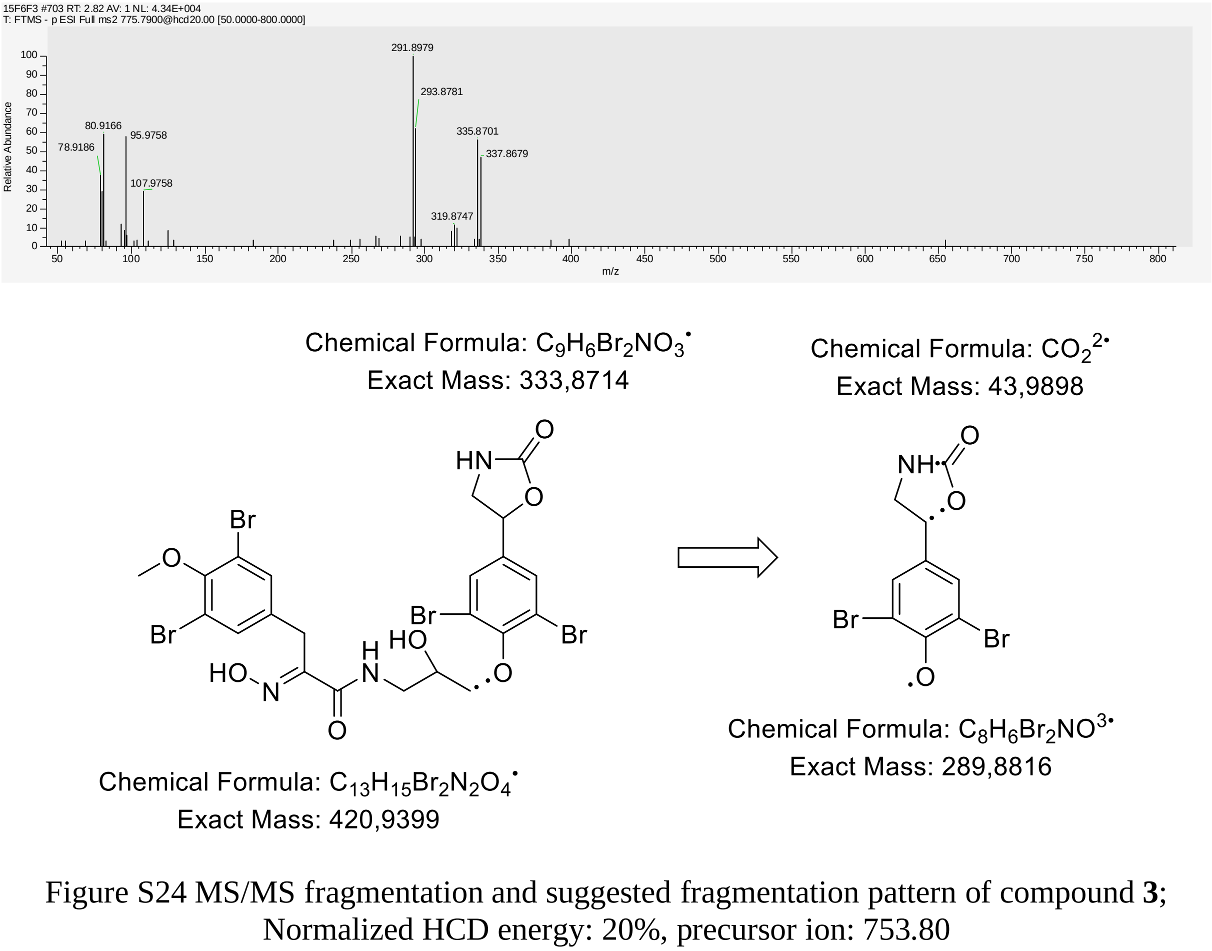

Figure S24 MS/MS fragmentation and suggested fragmentation pattern of compound **3**; Normalized HCD energy: 20%, precursor ion: 753.80

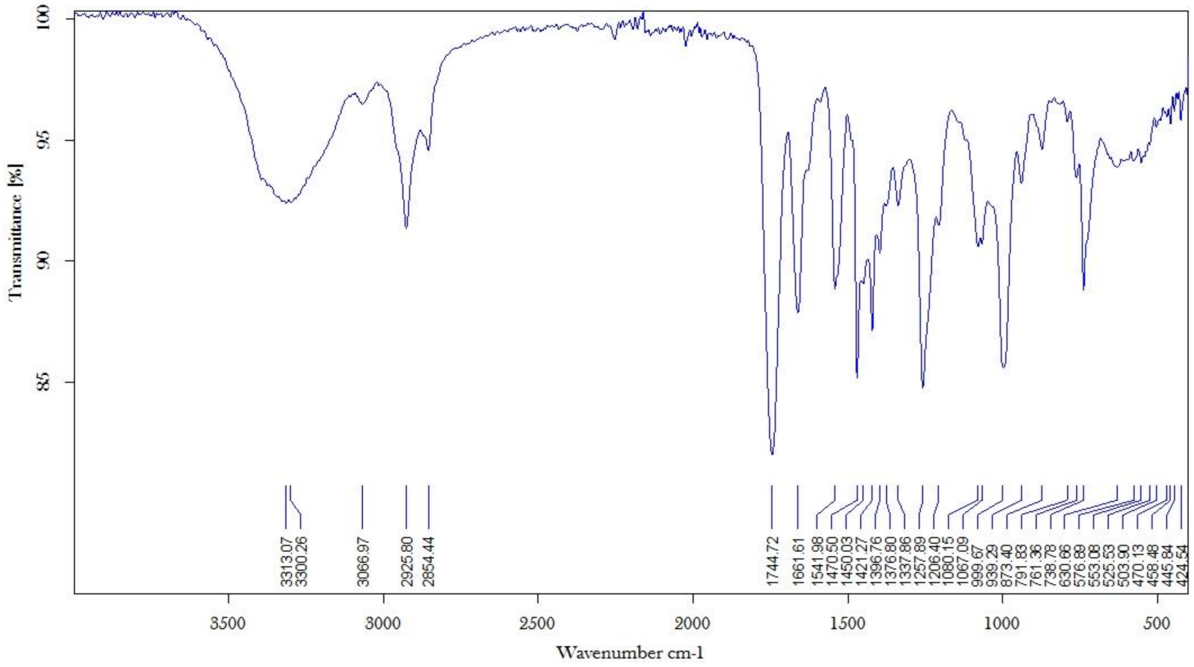

Figure S25. IR spectrum of compound **3**

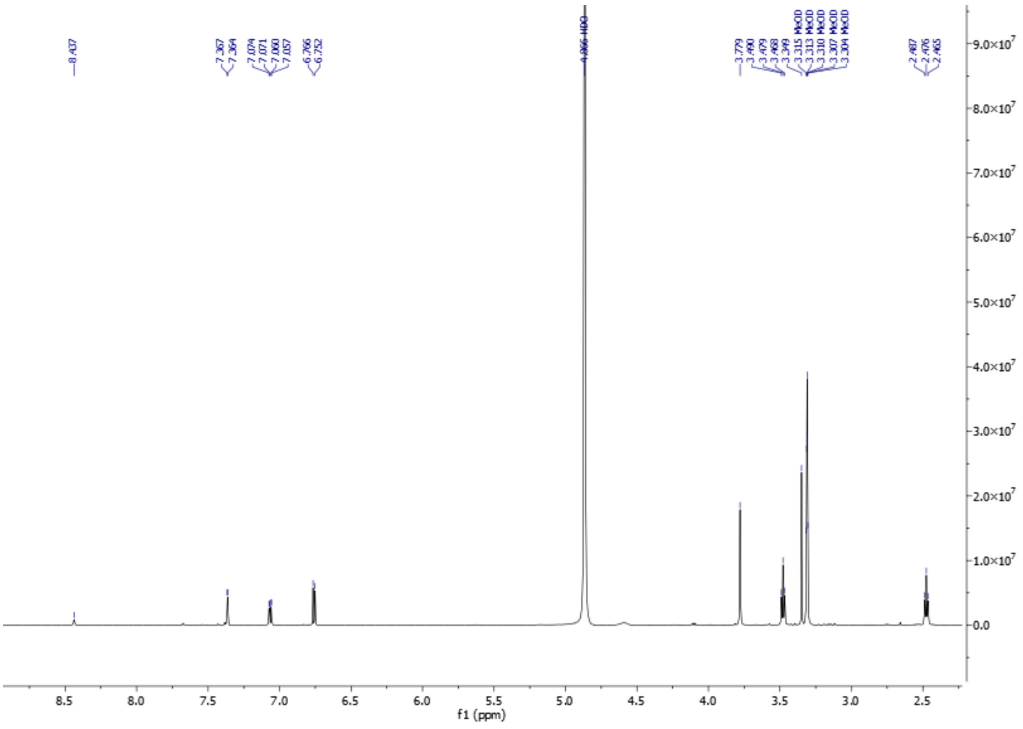

Figure S26. ^{1}H NMR spectrum of compound **4** in MeOH

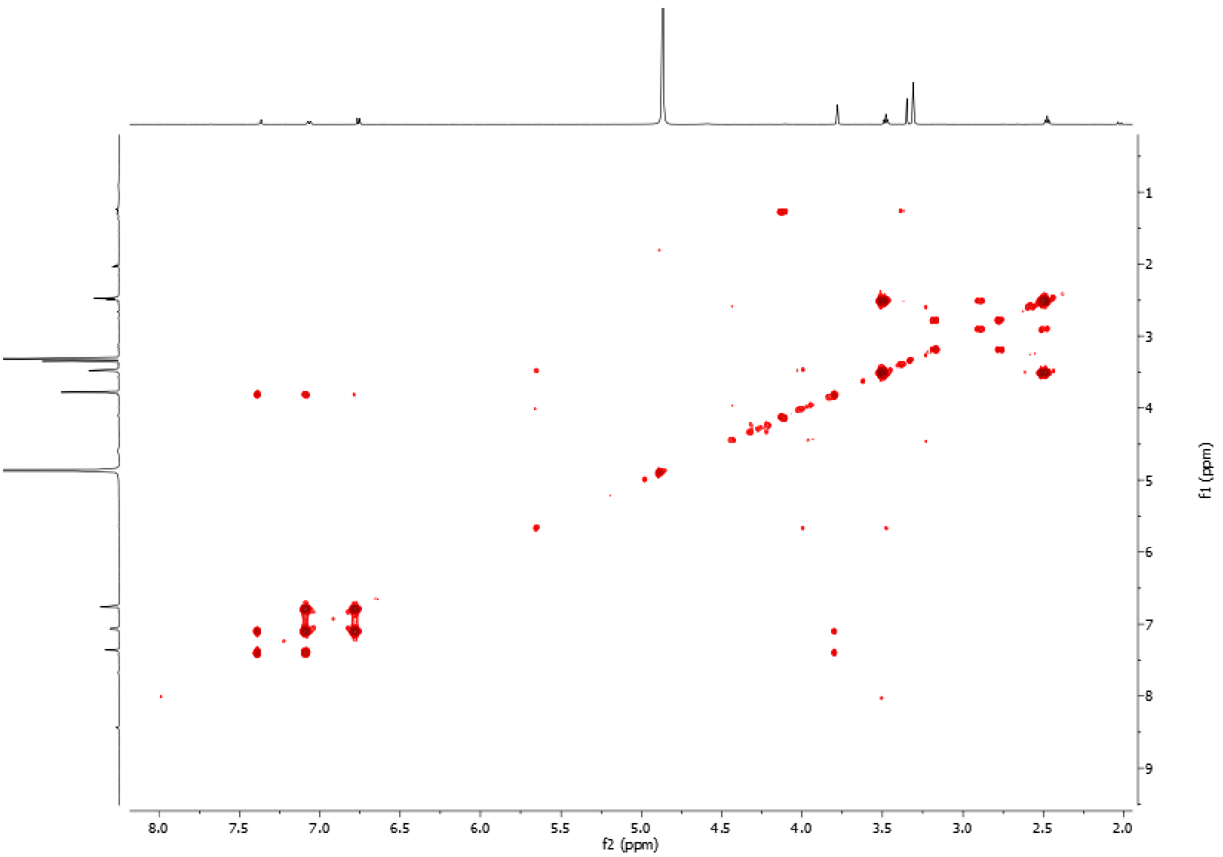

Figure S27. COSY spectrum of compound **4** in MeOH

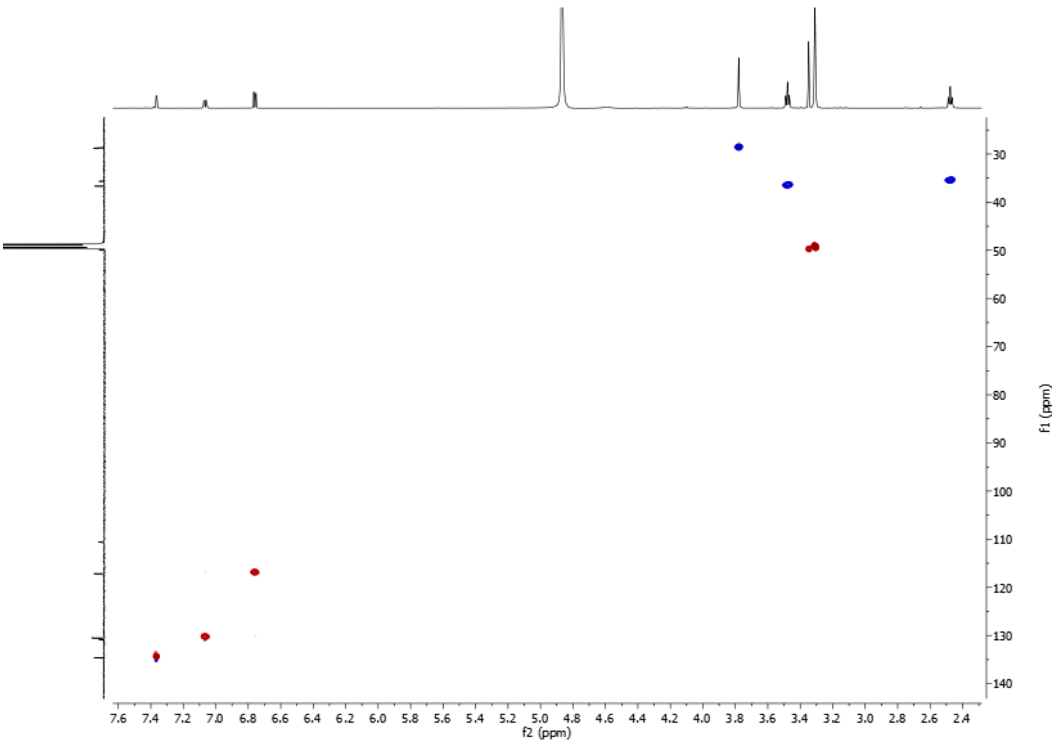

Figure S28. HSQC spectrum of compound **4** in MeOH

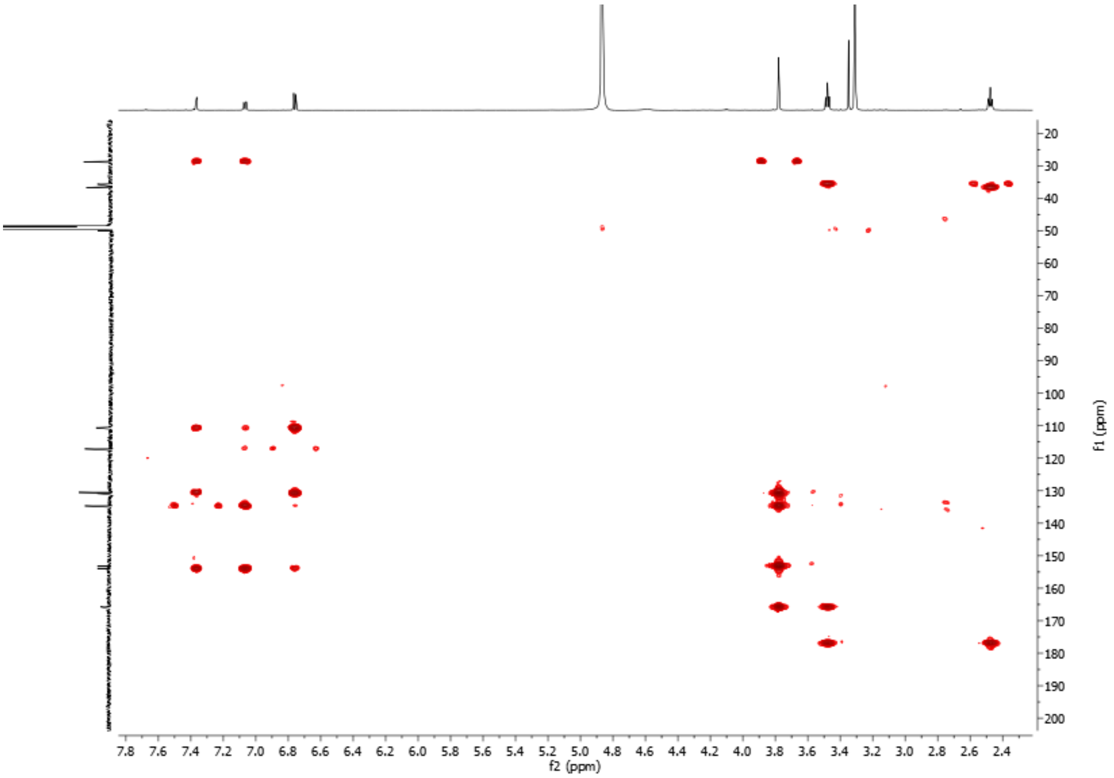

Figure S29. HMBC spectrum of compound **4** in MeOH

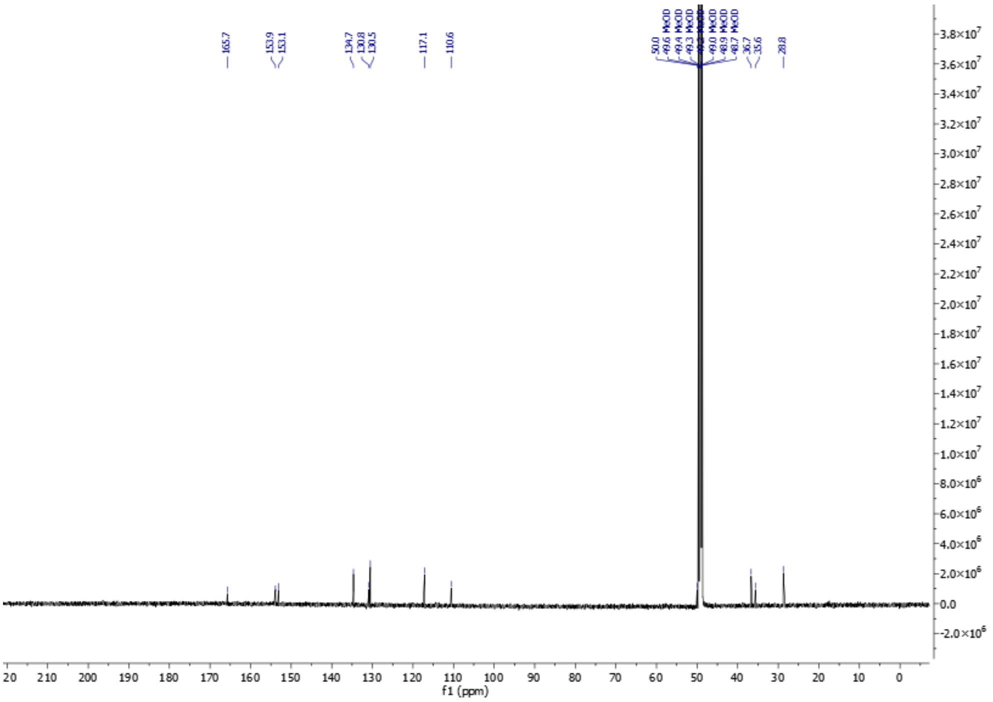

Figure S30. ^{13}C NMR spectrum of compound **4** in MeOH

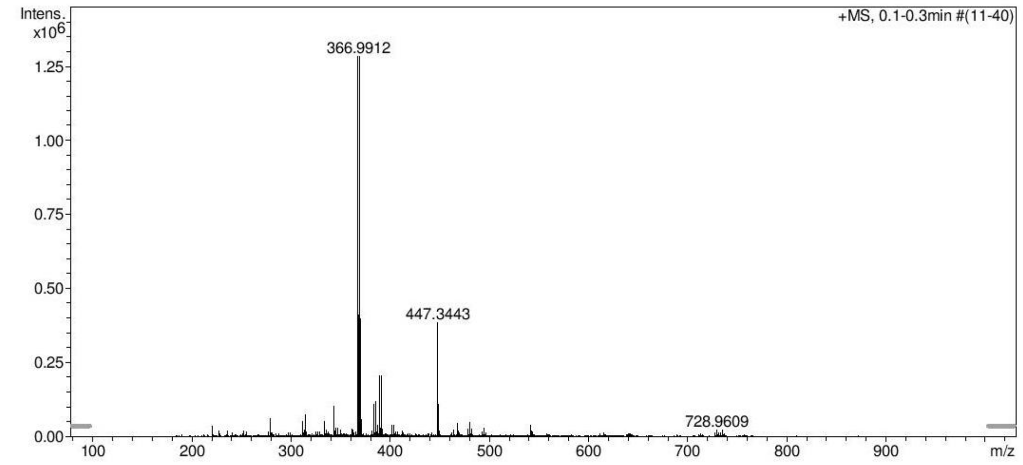

Figure S31. High-resolution mass spectrum of compound **4**

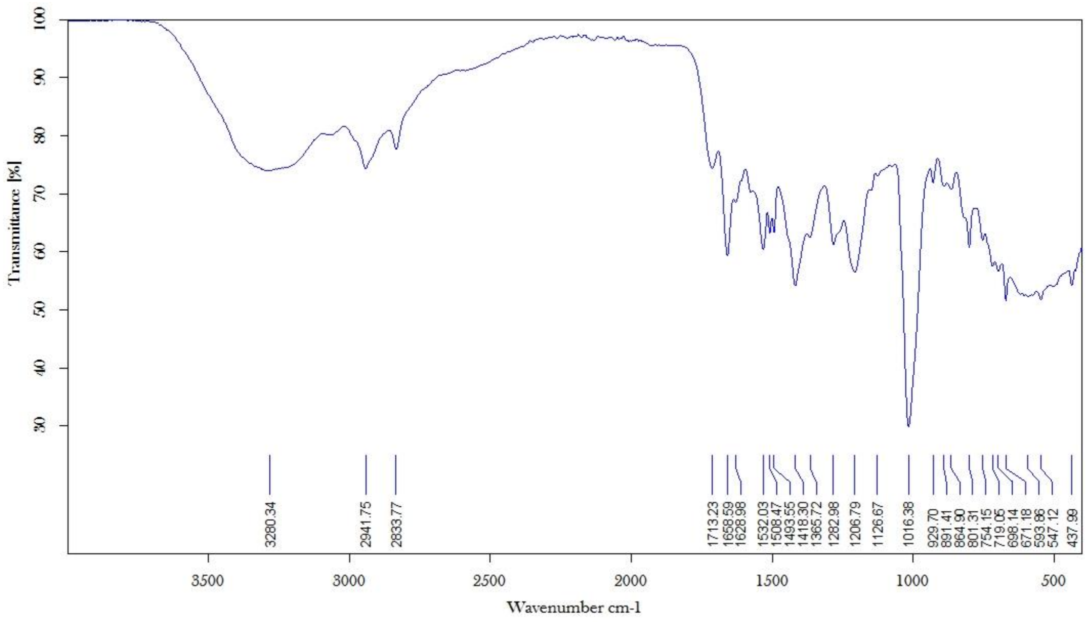

Figure S32. IR spectrum of compound **4**

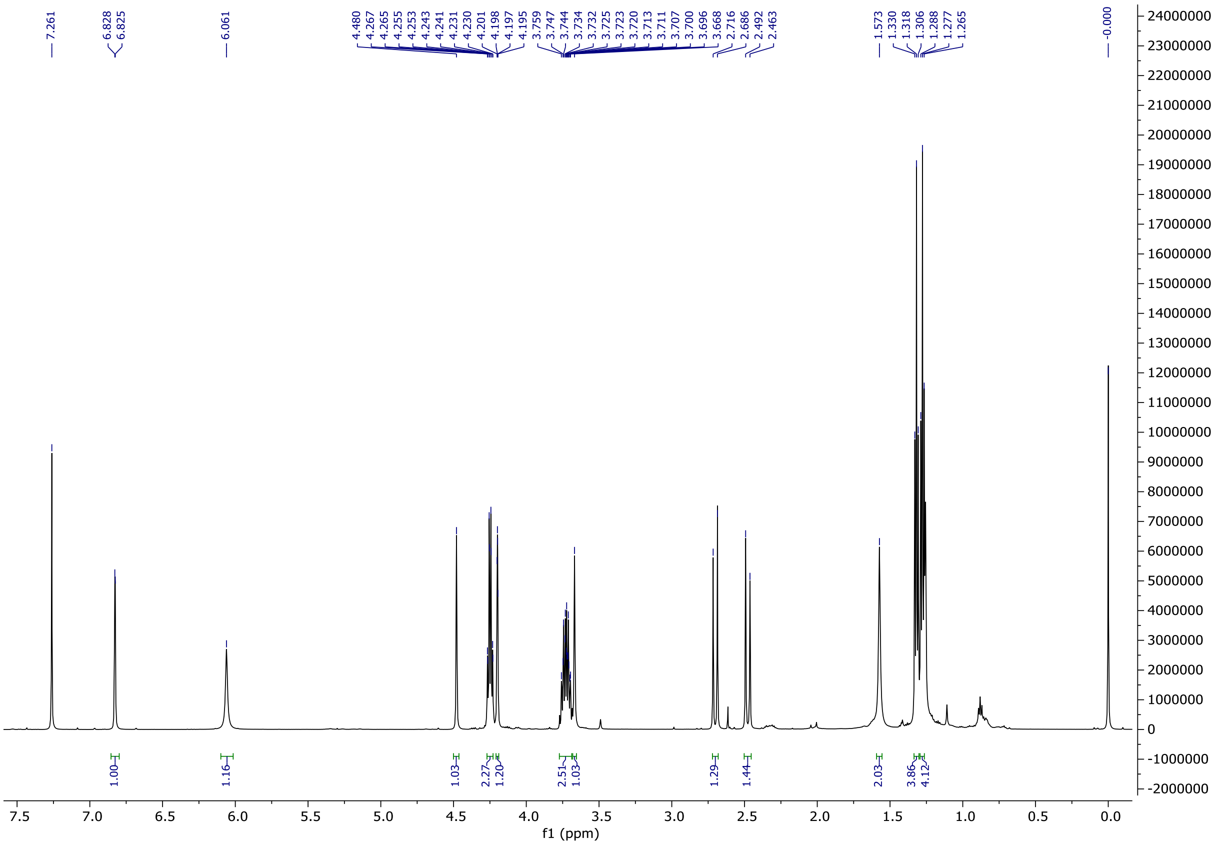

Figure S33. ^{1}H NMR spectrum of compound **5** in $CHCl_3$

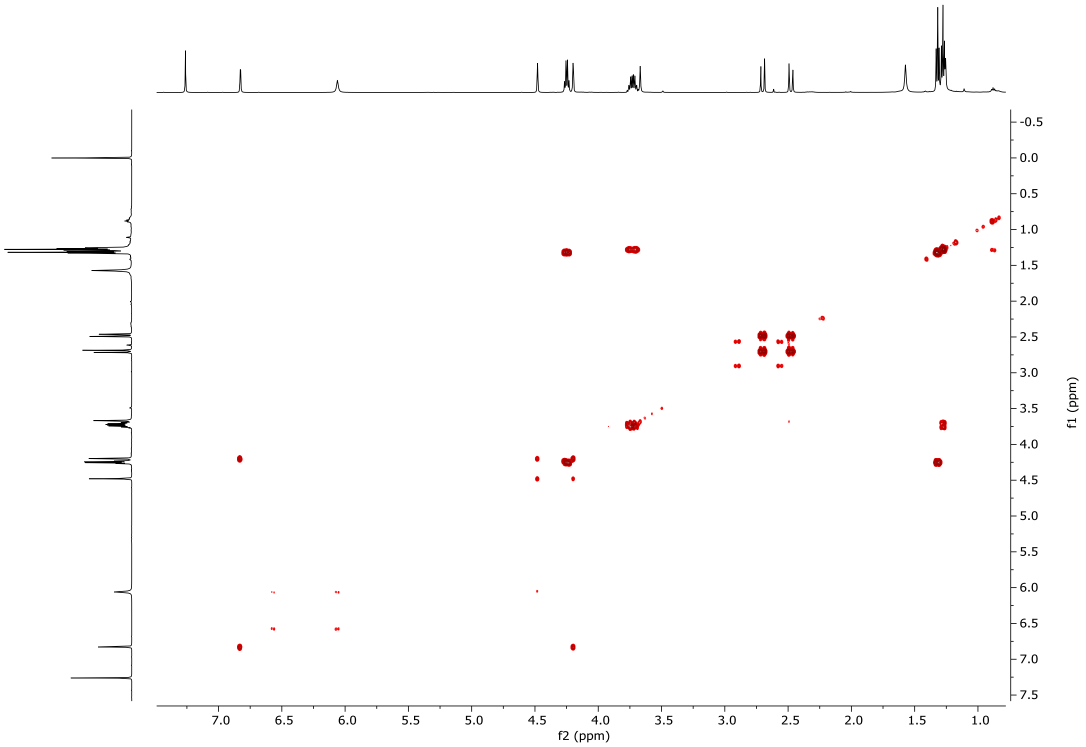

Figure S34. COSY spectrum of compound **5** in $CHCl_3$

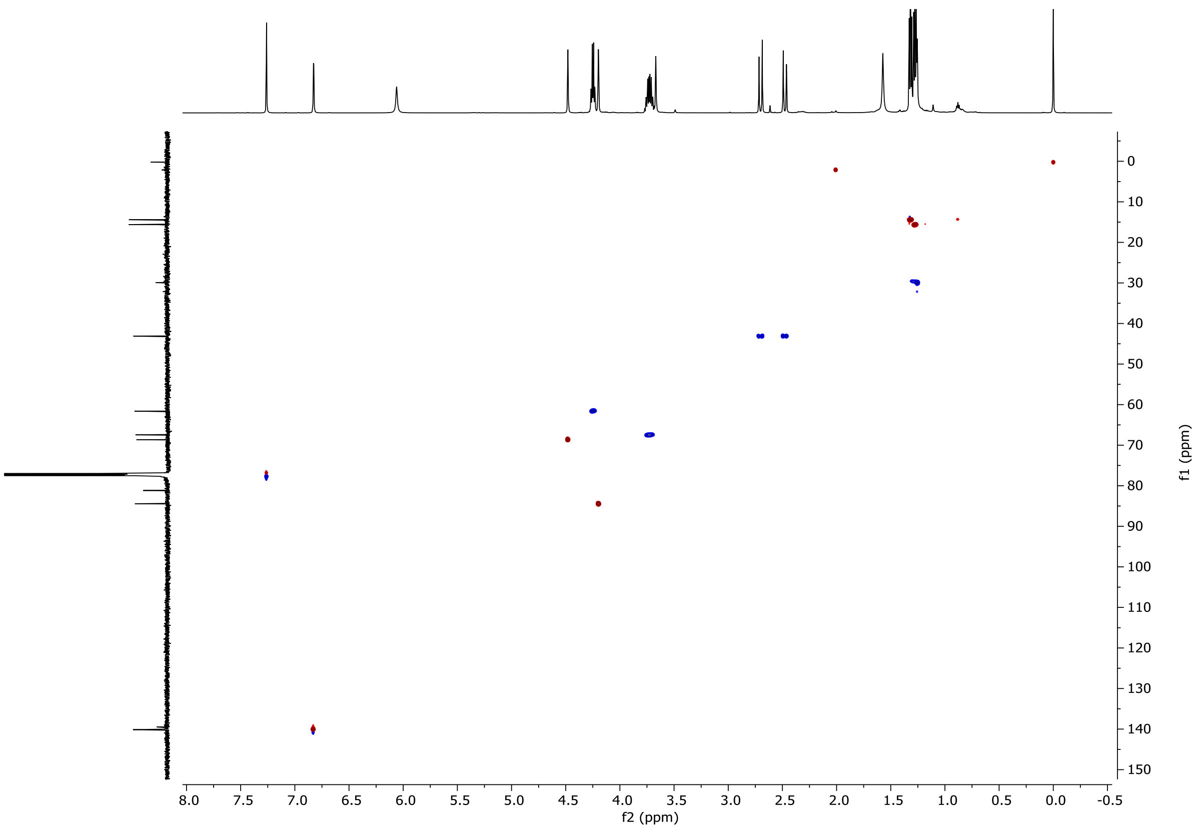

Figure S35. HSQC spectrum of compound **5** in $CHCl_3$

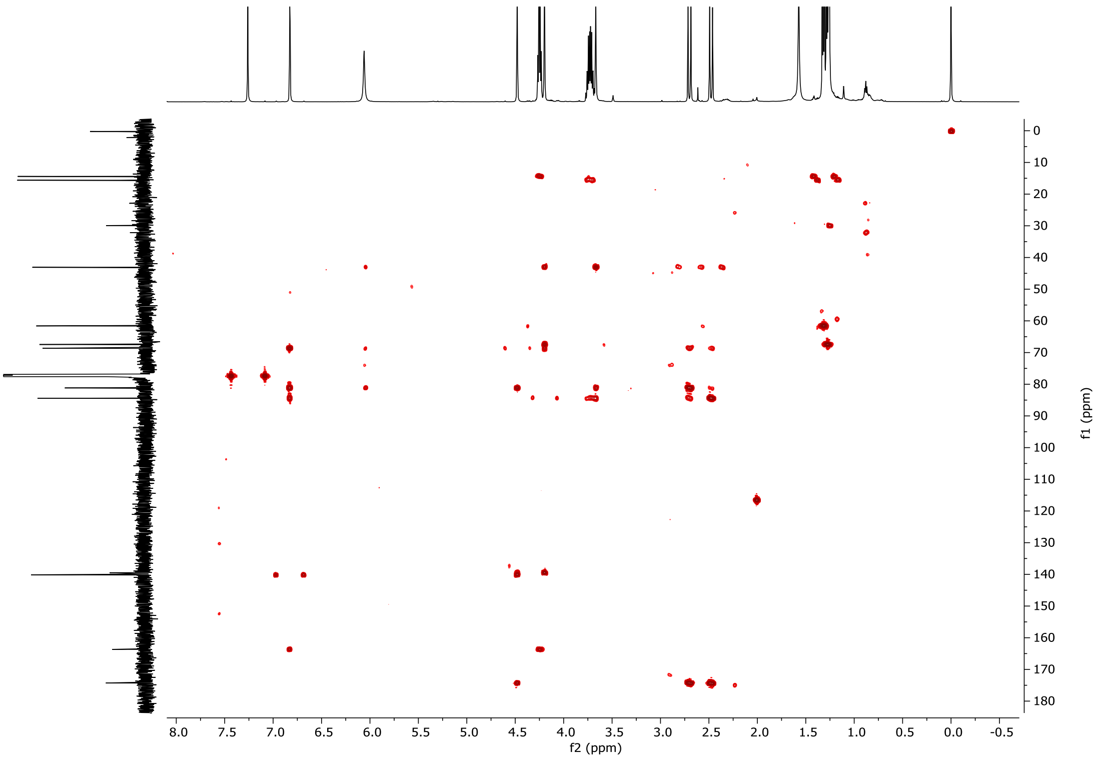

Figure S36. HMBC spectrum of compound **5** in $CHCl_3$

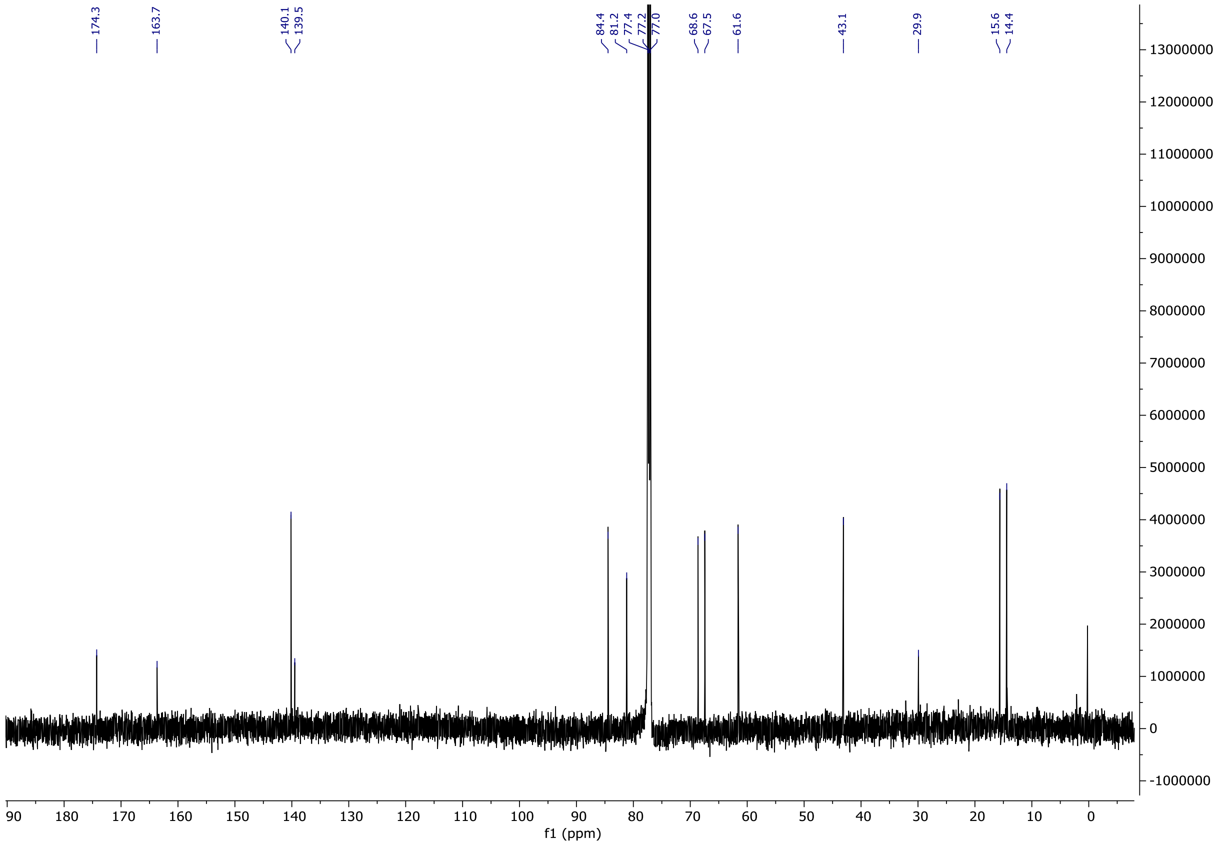

Figure S37. ^{13}C NMR spectrum of compound **5** in $CHCl_3$

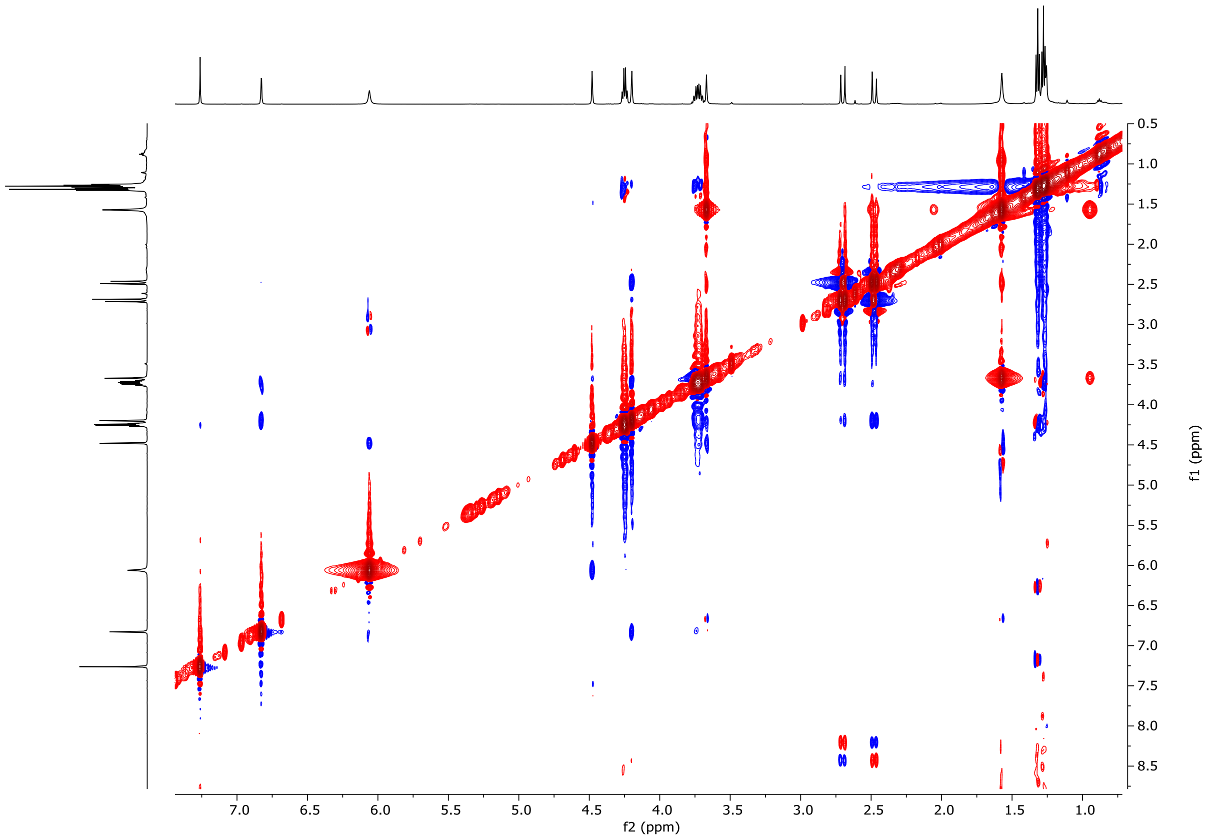

Figure S38. NOESY spectrum of compound **5** in $CHCl_3$

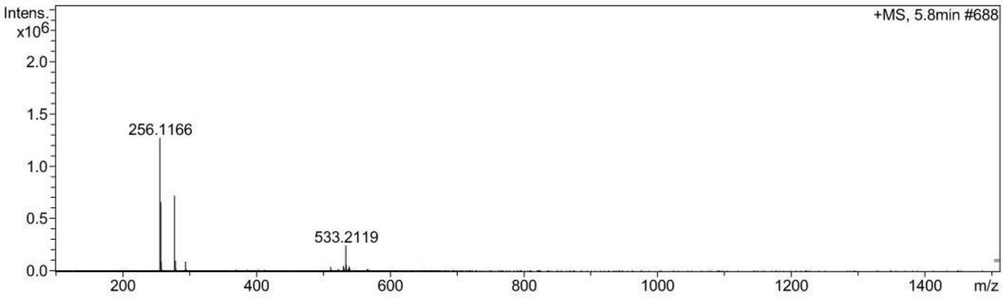

Figure S39. High-resolution mass spectrum of compound **5**

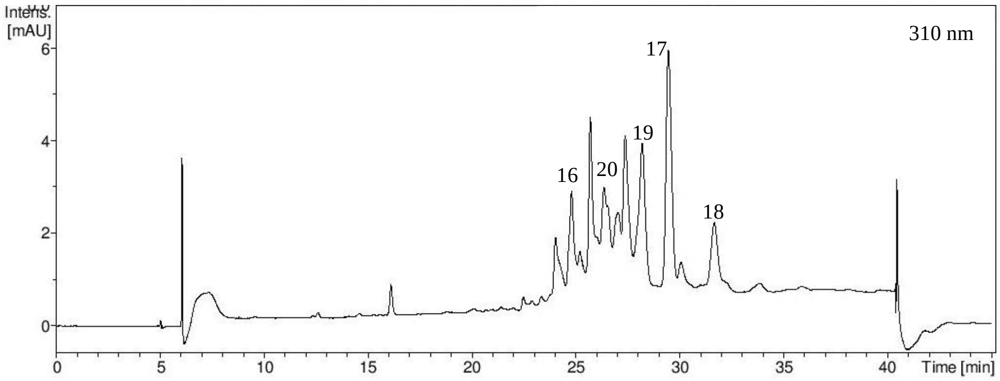

Figure S40. HPLC separation of the *Spongia* spp. extract. Peak assignment is according to figure 1. column: Synergi Polar-RP 80A (250 × 4, 6mm; 4 μm) from Phenomenex (Torrance, CA, USA); mobile phase: 0.1% (v/v) formic acid in water (A) and CH3CN (B); Gradient: 0 min: 2% B, 2 min: 50% B, 18 min: 90% B, 30-35 min: 98% B, 35.1-45 min: 2% B; λ= 210, 254, 280, 310 and 350 nm; flow rate=0.7 mL/min; T=22°C.

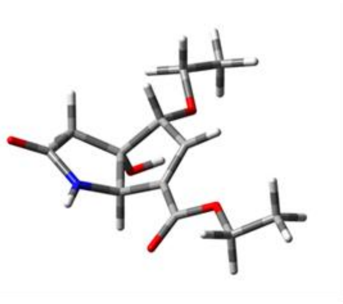

C1, 40.34%, -56.63

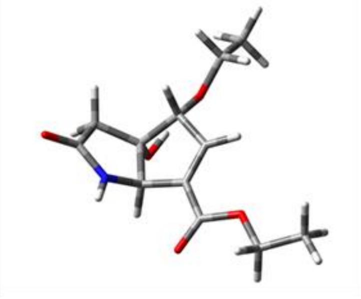

C2, 19.14%, -68.61

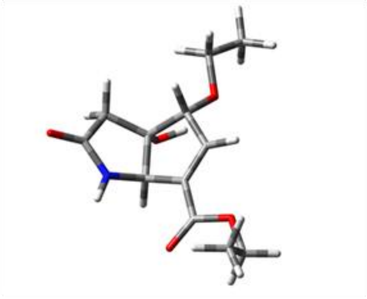

C3, 10.38%, -40.58

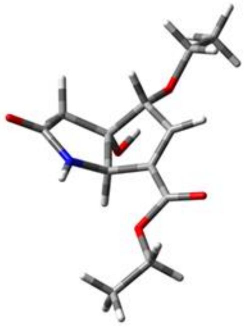

C4, 9.24%, -64.72

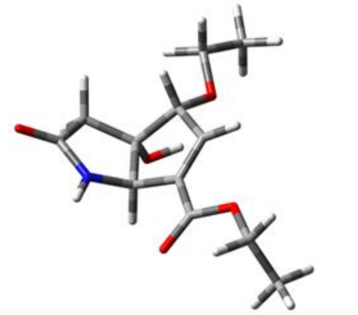

C5, 4.78%, -53.0

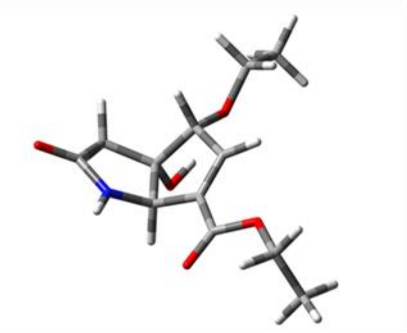

C6, 4.41%, -78.87

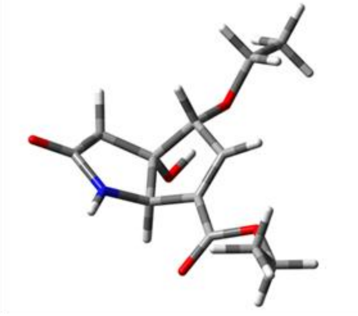

C7, 4.40%, -46.83

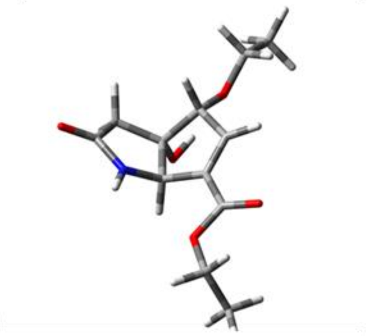

C8, 3.79%, -96.96

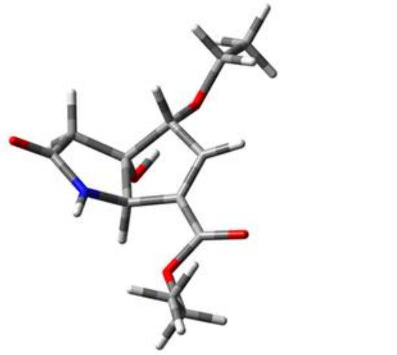

C9, 3.54%, -50.35

Figure S41. The most stable conformers (population > 3%) of compound **5** optimized at B3LYP/6-31G++(d,p)/CPCM/chloroform level and the calculated specific rotation values of each conformer at B3LYP/aug-pVTZ/CPCM/chloroform level using frequency of sodium D line (589.3 nm).

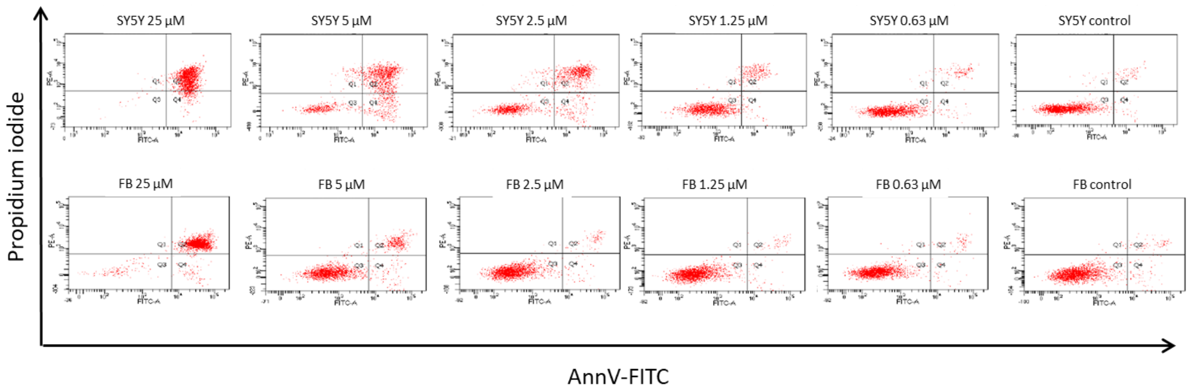

Figure S 42: SH-SY5Y cells and fibroblasts (FB), respectively, were treated with different concentrations of compound 13 (25 µM – 0.63µM) or left untreated (control). A representative analysis is shown. Cells alive are displayed in the lower left quadrant (Q3), early apoptotic cells are shown in Q4 (lower right), late apoptotic cells appear in the upper right quadrant (Q2) and necrotic cells would appear in the upper left quadrant (Q1).

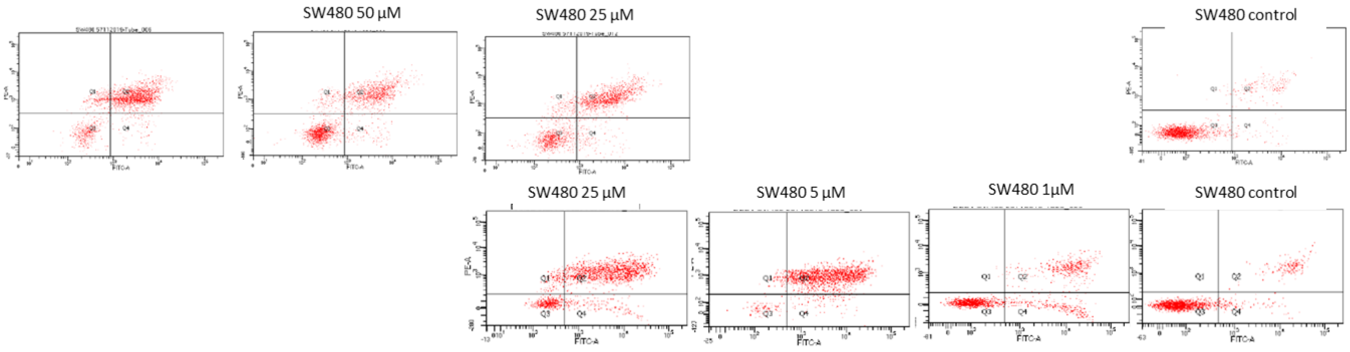

Figure S 43: SW480 colon carcinoma cells were treated with different concentrations of compound 13 (100 µM – 25 µM) or left untreated (control). A representative analysis is shown in the upper row. In the lower row a representative experiment using different concentrations (25 µM-1 µM and untreated control) is displayed. Cells alive are displayed in the lower left quadrant (Q3), early apoptotic cells are shown in Q4 (lower right), late apoptotic cells appear in the upper right quadrant (Q2) and necrotic cells would appear in the upper left quadrant (Q1).
